# Research progress on protein lactylation in female reproductive disease: molecular mechanisms, functions, and therapeutic implications

**DOI:** 10.3389/fphar.2026.1867863

**Published:** 2026-09-09

**Authors:** Xiaoyang Shen, Yan Chen, Hongxia Deng, Nannan Zhang, Liangzhi Xu

**Affiliations:** 1 Reproductive Endocrinology and Regulation Laboratory, West China Second University Hospital, Sichuan University, Chengdu, China; 2 Department of Obstetrics and Gynecology, West China Second University Hospital, Sichuan University, Chengdu, China; 3 Key Laboratory of Birth Defects and Related Diseases of Women and Children (Sichuan University), Ministry of Education, Chengdu, China; 4 National Center for Birth Defect Monitoring, West China Second University Hospital, Sichuan University, Chengdu, China

**Keywords:** epigenetic regulation, female reproductive diseases, gynecological tumors, lactate, lactylation, post-translational modification, therapeutic target

## Abstract

Female reproductive health depends on precisely coordinated metabolic, hormonal, immune, and epigenetic networks. Lactylation is a lactate-derived lysine post-translational modification that provides a direct mechanism by which glycolytic metabolism influences chromatin activity and protein function. Lactylation exerts diverse biological effects across multiple systems, including the cardiovascular, musculoskeletal, nervous, and urinary systems. Although individual studies have indicated that lactylation participates in key reproductive processes and contributes to various female reproductive disorders, the mechanisms connecting lactylation with female reproductive health and disease have not been systematically reviewed. Thus, we provide an integrated overview of lactylation-associated cellular mechanisms and the research progress in female reproductive health and disease. This review examines the molecular mechanisms of lactylation and its interactions with other post-translational modifications. We further summarize the physiological and pathological functions of lactylation in the female reproductive system, with particular emphasis on its context-dependent and double-edged role. We also outline emerging therapeutic strategies targeting lactate production, transport, lactylation enzymes, specific lactylation sites, together with clinical trials, while emphasizing key challenges associated with tissue specificity, reproductive safety, and precise modulation. This review highlights lactylation as a metabolic-epigenetic regulator and a potential therapeutic target for precision management of female reproductive diseases.

## 1 Introduction

In the 1920s, Otto Warburg first proposed the “Warburg effect”, which states that cancer cells preferentially produce adenosine triphosphate (ATP) through glycolysis even in aerobic environments, resulting in high lactate levels (Hanahan et al., 2011; Hanahan, 2022). Lactate was thought to be a metabolic waste product until the discovery of histone lysine lactylation in 2019 established a direct biochemical connection between glycolytic metabolism and gene transcription (Zhang et al., 2019). Lactylation is a lactate-derived post-translational modification (PTM) in which lactyl groups are added to lysine residues on histone and non-histone proteins, thereby influencing chromatin state, transcriptional activity, protein stability, enzymatic function, and intracellular signaling (Zhang et al., 2019; Chen J. et al., 2025; Shi et al., 2025). Increasing evidence indicates that lactylation influences diverse biological processes, including embryonic development, metabolic reprogramming, and inflammation (Ding et al., 2024; Zhang C. et al., 2025). The regulation of lactylation relies on a coordinated system of enzymes comprising “writers”, “erasers”, and “readers” (Shi et al., 2025; Sui et al., 2025). Aberrant regulation of protein lactylation has been connected to the pathogenesis of several diseases, including diabetes, myocardial infarction, immune dysregulation, and cancers (Chen J. et al., 2025; Wei et al., 2024a; Yang Z. et al., 2024; Chen X. et al., 2020; Wang Z. M. et al., 2025).

The female reproductive system, comprising the ovaries, fallopian tubes, uterus, and vagina, is essential for women’s reproductive ability and health (Sun X. et al., 2024). Its normal function depends on a complex and highly dynamic microenvironment characterized by coordinated interactions among germ cells, granulosa cells, stromal cells, and various immune cells (Wu et al., 2004). Reproductive system disorders, including gamete abnormalities, reproductive endocrine disorders, gestational diseases, and tumors, have become a major global public issue affecting reproductive health (Gao Y. et al., 2025; Huang et al., 2025). These conditions not only cause infertility and pregnancy complications but are also associated with broader long-term health implications that extend to both mothers and offspring (Nichols et al., 2024; Stener-Victorin and Deng, 2025). The pathogenesis of these disorders involves heterogeneous mechanisms, including hormonal dysregulation, chronic inflammation, oxidative stress, and defective programmed cell death, which together account for the current lack of effective preventive strategies against the onset and progression (Binmahfouz, 2026).

Lactate metabolism and protein lactylation have been shown to exert profound effects on reproductive tissues, particularly within the ovarian microenvironment and the uterine maternal–fetal interface (Yang Q. et al., 2022; Liu K. et al., 2024). Recent studies further indicate that lactylation is involved in the regulation of oocyte development, early embryonic development, endometrial receptivity, inflammatory responses, and tumor progression (Yang Q. et al., 2022; Sun J. et al., 2024; Wu et al., 2024; Zhao et al., 2024a). Moreover, unlike many other organ systems, the reproductive system can be investigated using experimentally accessible *ex vivo* models, including follicle and embryo cultures as well as endometrial organoids (Dong et al., 2025). These models provide unique platforms for defining stage-specific lactate–lactylation signaling during reproductive development and disease. From a clinical perspective, this *ex vivo* accessibility may facilitate assisted reproductive technology (ART) optimization and provide new opportunities for improving infertility treatment. Therefore, elucidating the mechanism of lactylation in the pathogenesis of female reproductive disorders is crucial for enhancing fertility, supporting healthy pregnancies, and preserving comprehensive gynecological health.

Here, we provide a reproduction-focused review of protein lactylation, covering the molecular basis of lactate metabolism and lactylation, and its crosstalk with other PTMs. We highlight recent advances in the identified lactylation sites and the regulatory mechanisms in the physiology and disease states of the female reproductive system. We systematically review current therapeutic targets for lactylation and address current challenges and future perspectives. Compared with the recent review on the enzymatic mechanisms of lactylation in gynecological diseases, our review emphasizes female reproductive biology and translational pharmacology, providing a more systematic and comprehensive synthesis of disease-specific evidence, therapeutic strategies, and clinical translation in female reproductive disorders (Zhang M. et al., 2026). This review provides a conceptual foundation and direction for advancing subsequent investigation and the clinical application of protein lactylation in reproductive health.

## 2 Molecular mechanism of lactylation

### 2.1 Lactate and lactylation

#### 2.1.1 Production of lactate

Lactate, the direct donor of the lactyl group and the direct trigger of protein lactylation, is mainly generated through glycolysis. In the cytoplasm, glucose is converted to pyruvate, which enters the mitochondria under normal aerobic conditions for oxidation through the tricarboxylic acid (TCA) cycle. Under hypoxic conditions or increased metabolic demand, however, pyruvate is reduced to L-lactate by lactate dehydrogenase A (LDHA) with hydrogen atoms provided by reduced nicotinamide adenine dinucleotide (NADH), while regenerating nicotinamide adenine dinucleotide (NAD^+^) to sustain glycolysis (Xiao et al., 2025). In contrast, lactate dehydrogenase B (LDHB) can catalyze the reverse conversion of lactate to pyruvate under more oxidative conditions (Rabinowitz and Enerbäck, 2020). Pyruvate is then converted to acetyl-CoA via the pyruvate dehydrogenase complex (PDC) in the mitochondria, participating in the TCA cycle for aerobic oxidation, and producing ATP (Rabinowitz and Enerbäck, 2020). In addition to glucose, cancer cells can utilize glutamine as their second major carbon source, generating lactate to provide energy for cell growth and proliferation (Chen J. et al., 2025).

As a common metabolic byproduct in cellular microenvironments, lactate is an important energy-supporting substrate, and it facilitates energy transfer between different cell types via the lactate shuttle mechanism. Lactate that accumulates in tumors due to the Warburg phenomenon can be converted to pyruvate and subsequently enter the TCA cycle as an oxidative substrate to meet the metabolic needs of cancer cells and establish conditions that favor tumor progression (Rho and Hay, 2025). This metabolic shift not only supports cellular energy and biosynthetic demands but also creates a lactate-rich microenvironment favorable for signal transduction and epigenetic remodeling. The activity of LDHA is modulated by a suite of upstream transcription factors, with hypoxia-inducible factor (HIF) being particularly prominent under hypoxic conditions (Silagi et al., 2021). HIF-1α promotes lactate production by upregulating glucose transporters and glycolytic enzymes. It also induces pyruvate dehydrogenase kinase 1 (PDK1) expression and inhibits pyruvate dehydrogenase (PDH). This results in the prevention of pyruvate from entering the TCA cycle and instead redirects it towards lactate synthesis (Silagi et al., 2021; Luo et al., 2011).

#### 2.1.2 Lactate transportation

The equilibrium between lactate generation and clearance is maintained by transmembrane proteins monocarboxylate transporters (MCTs), among which MCT1 and MCT4 are the best characterized (Ji and Xia, 2025). MCT1 is widely expressed in various tissues and operates as a bidirectional transporter. Whereas MCT4 is primarily found in cells with high glycolytic activity, such as white skeletal muscle, astrocytes, and white blood cells, and is mainly responsible for lactate efflux (Xiao et al., 2025; Ji and Xia, 2025). These transporters mediate H^+^-coupled lactate exchange across the plasma membrane, thereby contributing to intracellular pH homeostasis, extracellular acidification, and metabolic communication between neighboring cells (Chen J. et al., 2025). Lactate transport via MCTs also participates in signal transduction and intercellular communication, such as the cellular exchanges in the decidual microenvironment during early pregnancy, as well as the tumor microenvironment (Zuo et al., 2015; Huang C. et al., 2024).

#### 2.1.3 Lactate functions as a signaling molecule

Lactate exerts signaling functions by activating the transmembrane G protein-coupled receptor (GPR). The most well-characterized lactate receptor is GPR81, which acts as a primary sensor mediating lactate signaling (Liu X. et al., 2024). Activation of this receptor leads to the inhibition of adenylate cyclase activity, which reduces cyclic adenosine monophosphate (cAMP) levels and ultimately suppresses protein kinase A (PKA) activity (Ahmed et al., 2010; Ren H. et al., 2025). Tumor-derived lactate can also activate GPR81 on adjacent non-malignant cells in a paracrine manner, contributing to intercellular communication in the tumor microenvironment (Su et al., 2024). Through these autocrine and paracrine signaling mechanisms, GPR regulates key cancer-related processes, including metabolic reprogramming, immune evasion, epithelial-mesenchymal transition (EMT), angiogenesis, and tumor cell survival (Brown and Ganapathy, 2020).

#### 2.1.4 Lactate functions through lactylation

In addition to its roles in energy supply and signal transduction, lactate can modulate several cellular processes via lactylation, a recently identified PTM (Rho and Hay, 2025). In 2019, Zhao et al. first identified lactylation on histone lysine residues in human cancer cells and mouse macrophages (Zhang et al., 2019). The lactyl group, mainly derived from L-lactate, is characterized by its unique structure that includes a carbonyl group adjacent to a hydroxyl group (Zhang et al., 2019; Zhao W. et al., 2024). This structural arrangement facilitates its effective binding to the lysine residues and modulates protein function (Zhao W. et al., 2024).

**FIGURE 1:**
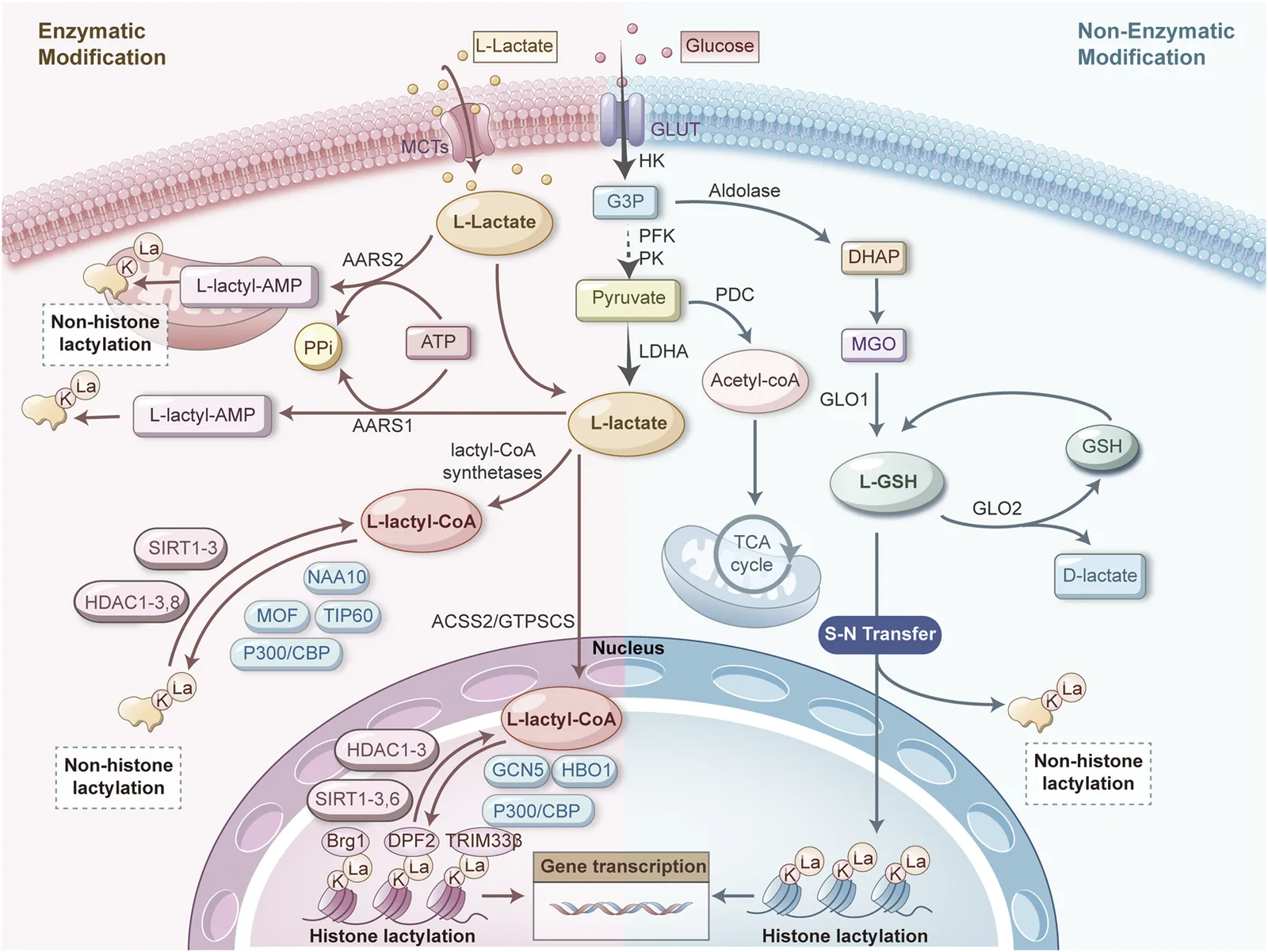
Cellular regulation of enzymatic and non-enzymatic lysine lactylation. Lysine lactylation occurs through enzymatic and non-enzymatic pathways. In the enzymatic pathway, L-lactate is either imported through MCTs or produced from pyruvate by LDHA, then converted to L-lactyl-CoA by ACSS2/GTPSCS for writer-mediated lactylation of histone and non-histone lysine residues. AARS1 and AARS2 can also generate L-lactyl-AMP from L-lactate and ATP to drive CoA-independent non-histone lactylation. These marks can be removed by erasers or recognized by readers to regulate chromatin remodeling, gene transcription, and protein function. In the non-enzymatic pathway, DHAP-derived MGO is converted by GLO1 and GSH into LGSH, which spontaneously transfers lactyl groups to lysine residues via an S-N transfer mechanism.

Lactylation can be classified as either enzymatic or non-enzymatic. Enzymatic lactylation involves two distinct mechanisms, one relying on lactyl-CoA and the other not. In the lactyl-CoA-dependent pathway, lactyl‐CoA synthetase transforms L-lactate into L-lactyl-CoA. Then the lactyltransferases (writers) catalyze the transfer of the lactyl group to a lysine residue. In the lactyl-CoA-independent pathway, alanyl-tRNA synthetases (AARS1/2) act as lactyltransferases. AARS first binds to lactate and forms L-lactyl-AMP and PPi in the presence of ATP; then, AARS transfers lactyl groups to lysine residues on target proteins (Zong et al., 2024; Chang et al., 2025). Readers are responsible for identifying and interpreting lactylation to send signals to downstream targets. Finally, lysine lactylation is reversible; erasers can remove L-lactyl groups from both histone and non-histone proteins (Peng and Du, 2025). Protein lactylation is dynamically regulated by a coordinated network of “writers”, “erasers”, and “readers”, which together determine the spatial and temporal patterns of this modification in response to metabolic signals (Figure 1).

In the non-enzymatic lactylation pathway, D-lactate reacts with glutathione (GSH) by glyoxalase 1 (GLO1) to form lactoylglutathione (LGSH), which functions as a donor of the lactyl group. LGSH is then hydrolyzed by glyoxalase 2 (GLO2), which recycles the glutathione while releasing D-lactate. The lactyl group can be transferred non-enzymatically from LGSH to a protein lysine residue via S-to-N transfer (Gaffney et al., 2020) (Figure 1). GLO2 downregulation leads to LGSH accumulation, resulting in D-lactylation of glycolytic enzymes, creating a negative feedback loop that reduces glycolytic flux (Gaffney et al., 2020). D-lactylation of RelA suppresses the transcriptional activity of nuclear factor-kappa B (NF-κB) and prevents excessive inflammation (Zhao Q. et al., 2025). Moreover, accumulated LGSH can promote site-specific histone D-lactylation, which potentiates the inflammatory response of macrophages (Trujillo et al., 2024). Thus, GLO2 is a promising therapeutic target for inflammatory disorders.

The distribution of lactylation encompasses a variety of tissue types, including the reproductive system organs such as the uterus and ovary, as well as non-reproductive tissues (Yang D. et al., 2024). Numerous histone lactylation modification sites, mostly on lysine residues, have been identified. Among these, H3K18la, H4K5la, H4K12la, and H4K18la have been studied most extensively (Sheng et al., 2025). In addition to histones, this modification extensively targets non-histone proteins in various subcellular compartments, including the cytoplasm, mitochondria, ribosomes, and cell membrane (Zhang et al., 2026a). Lactylation exerts its functions through various mechanisms, which can be broadly categorized into the following five types: (1) Epigenetic regulation: histone modifications influence chromatin structure and gene transcription (Sheng et al., 2025). (2) Regulation of protein function: lactylation alters protein structure, stability, enzymatic activity, and interactions with other molecules (Zhang Q. et al., 2025). (3) Cellular metabolism and energy homeostasis: modifications of metabolic enzymes or energy sensors modulate metabolic flux. (4) Cellular stress and homeostasis maintenance: involving redox balance, autophagy, lysosomal pathways, and DNA damage repair (Wu N. et al., 2025). (5) Cell fate and behavior: encompassing cell survival and death, cell proliferation, differentiation, and development (Ding et al., 2025). These properties make lactylation particularly relevant to the reproductive system, where metabolic status must be tightly coordinated with epigenetic regulation during gametogenesis, embryo development, implantation, placentation, and tumor progression. A deeper investigation into the biological mechanisms of lactylation will facilitate the development of novel diagnostic biomarkers and therapeutic targets.

### 2.2 The regulation of lactylation modification

#### 2.2.1 Key enzymes

##### 2.2.1.1 Key enzymes of the glycolytic pathway

The glycolytic pathway contains several key rate-limiting enzymes, primarily including hexokinase (HK), phosphofructokinase (PFK), and pyruvate kinase (PK) (Wang R. et al., 2024; Zhao R. et al., 2024; Bai et al., 2025) (Figure 1). These enzymes indirectly regulate protein lactylation by altering intracellular lactate levels, linking cellular metabolism to epigenetic reprogramming. In cancer cells, these enzymes are often abnormally activated, leading to the accumulation of lactate and the manifestation of the Warburg effect (Littleflower et al., 2024). The accumulated lactate serves as a substrate for lactylation, thereby promoting histone and non-histone protein lactylation. For example, upregulation of pyruvate kinase M2 (PKM2) promotes lactate accumulation and subsequent Snail1 lactylation, facilitating its nuclear translocation and driving EMT and metastasis in pancreatic cancer (Zhao R. et al., 2024).

##### 2.2.1.2 Lactate dehydrogenase

Lactate dehydrogenase is a central regulator of lactate homeostasis. LDHA primarily catalyzes the conversion of pyruvate to lactate (Figure 1). Under pathological conditions such as cancer, inflammation, or hypoxia, elevated LDHA expression promotes significant lactate accumulation and thereby facilitates lactylation (de la Cruz-López et al., 2019). LDHA is subject to multilayered regulation, including transcriptional regulation by factors such as HIF-1α, forkhead box protein M1 (FOXM1), and c-Myc, as well as PTMs, including phosphorylation, acetylation, and succinylation (Wang M. et al., 2025). Notably, LDHA itself undergoes AARS1-mediated lactylation, which enhances its enzymatic activity and establishes a positive feedback loop between lactate production and lactylation (Li J. et al., 2026).

##### 2.2.1.3 Lactyl-CoA synthetase

Lactyl-CoA synthetase catalyzes the reaction between lactate and CoA to generate the intermediate lactyl group donor. Recent studies have identified acetyl-CoA synthetase 2 (ACSS2) and guanosine triphosphate (GTP)-specific succinyl-CoA synthetase (GTPSCS) as nuclear enzymes capable of producing lactyl-CoA. ACSS2 can couple lactyl-CoA generation with KAT2A-mediated histone lactylation (Zhu R. et al., 2025), whereas GTPSCS can cooperate with p300 to enhance histone lactylation and activate downstream oncogenic transcriptional programs (Liu R. et al., 2025) (Figure 1).

##### 2.2.1.4 Lactyltransferases (writers)

The currently identified “writers” of lactylation are mainly lysine acetyltransferases and a small number of noncanonical lactyltransferases. They can utilize lactyl-CoA as a lactyl donor to catalyze the lactylation of lysine residues. There are three major families of lysine acetyltransferases (KATs): GNAT (KAT2A, KAT2B, HAT1), p300/CREB-binding protein (CBP) (KAT3), and MYST (KAT5, KAT6A, KAT7, KAT8) (Li K. et al., 2025) (Table 1, Figure 1). Histone lactylation neutralizes the positive charge of histones, which weakens the interaction with negatively charged DNA, and leads to an open chromatin conformation that facilitates gene transcription (Liu Y. et al., 2024). Among these, p300/CBP has emerged as the primary histone lactyltransferase, catalyzing lactylation at sites such as H3K9, H3K18, and H4K12 (Zhang et al., 2019; Sheng et al., 2025; Patel et al., 2020; Dai et al., 2023; Zhang and Zhang, 2024; Yang C. et al., 2025). Intracellular lactate accumulation can directly induce p300/CBP activity, thereby elevating histone lactylation levels (Wang L. et al., 2023). KATs also regulate non-histone lactylation, underscoring their expanded role in metabolic signaling and protein regulation (Chen Y. et al., 2024).

**TABLE 1:** Writers, erasers, and readers of lactylation.

Types	Regulator	Type	Localization	Modification site		Ref
				Histones	Non-histones	
Writers	p300/CBP	Lysine acetyltransferase	Nucleus	H3K9 H3K18 H4K12	MRE11 (K673)	Zhang et al. (2019), Dai et al. (2023), Zhang and Zhang (2024), Yang C. et al. (2025), Chen Y. et al. (2024)
	GCN5 (KAT2A)	Lysine acetyltransferase	Nucleus	H3K9 H3K14 H3K18 H4K12	RAD51 (K73)	Zhu R. et al. (2025), Sun et al. (2025), Wang et al. (2022), Chen M. et al. (2025)
	TIP60 (KAT5)	Lysine acetyltransferase	Cytoplasm Nucleus	​	Vps34 (K781/K356), NBS1 (K388)	Chen H. et al. (2024), Jia et al. (2023)
	HBO1 (KAT7)	Lysine acetyltransferase	Nucleus	H3K9 H3K18 H4K8	​	Niu et al. (2024), Zhang K. et al. (2025)
	MOF (KAT8)	Lysine acetyltransferase	Nucleus	H4K12 H3K9	eEF1A2 (K408), LTBP1 (K752)	Xie et al. (2024), Zou et al. (2024), Dai et al. (2025), Zou Y. et al. (2025)
	NAA10	Lysine acetyltransferase	Nucleus	​	NSUN2 (K508)	Niu et al. (2025)
	AARS1	Non-classical lactyltransferase	Cytoplasm	​	p53 (K120/K139)	Zong et al. (2024), Ju et al. (2024)
	AARS2	Non-classical lactyltransferase	Mitochondrion matrix	​	PDHA1 (K336), CPT2 (K457/8)	Mao et al. (2024)
Erasers	SIRT1	NAD^+^ dependent deacetylase	Nucleus and cytoplasm	H3K18	IRF9 (K81), Ran (K123)	Miao et al. (2026), Tsukihara et al. (2025), Wang et al. (2026)
	SIRT2	NAD^+^ dependent deacetylase	Nucleus and cytoplasm	H3K18 H4K8 H4K12	METTL16 (K229), USP2 (K447)	Sun et al. (2023), Zu et al. (2022), Xiang et al. (2025)
	SIRT3	NAD^+^ dependent deacetylase	Mitochondrion matrix	H4K16 H3K9	cyclin E2 (K348)	Jin et al. (2023), Fan et al. (2023), Chen C. et al. (2025)
	SIRT6	NAD^+^ dependent deacetylase	Mitochondrion matrix	H3K9 H3K18	TBK1 (K241)	Zou L. et al. (2025), Nickel et al. (2025), Xie et al. (2026)
	HDAC1	Class I histone deacetylase	Nucleus	H3K18 H4K5	​	Moreno-Yruela et al. (2022)
	HDAC2	Class I histone deacetylase	Nucleus and cytoplasm	H3K18 H4K5	​	Moreno-Yruela et al. (2022), Li F. et al. (2024)
	HDAC3	Class I histone deacetylase	Nucleus and cytoplasm	H3K18 H4K5	NBS1 (K388)	Moreno-Yruela et al. (2022), Chen H. et al. (2024)
	HDAC8	Class I histone deacetylase	Nucleus and cytoplasm	​	PRMT1 (K134/K145)	Zhou et al. (2025)
Readers	BRG1	Bromodomain‐containing protein	Nucleus	H3K18	​	Hu et al. (2024)
	DPF2	PHD finger domain‐containing protein	Nucleus	H3K14	​	Zhai et al. (2024)
	TRIM33β	PHD–bromodomain‐containing protein	Nucleus	H3K18	​	Nuñez et al. (2024)

In addition to classical acetyltransferases, noncanonical writers have recently been reported. N-alpha-acetyltransferase 10 (NAA10), a catalytic subunit of the N-terminal acetyltransferase complex, has been shown to function as a lactyltransferase (Magin et al., 2016; Niu et al., 2025). Recently, alanyl-tRNA synthetases AARS1 and AARS2 have been identified as the intracellular lactate sensors and lysine lactyltransferases, catalyzing a two-step ATP-dependent reaction that directly conjugates lactate to protein lysine residues (Li H. et al., 2024). AARS1 lactylates p53 to inactivate p53 and promote tumorigenesis (Zong et al., 2024), highlighting AARS1 as a key oncogenic regulator.

##### 2.2.1.5 Delactylases (erasers)

The two groups of verified protein delactylases are the classical zinc-dependent histone deacetylase (HDAC) and the NAD^+^-dependent sirtuin (SIRT) family (Table 1, Figure 1). Among them, Class I HDACs (HDAC1–3) have been conclusively identified as core histone delactylases and efficiently remove marks such as H3K18la and H4K5la (Moreno-Yruela et al., 2022). HDAC3 can also erase non-histone lactylation, indicating that HDAC-mediated delactylation is not restricted to chromatin substrates (Chen H. et al., 2024). Beyond Class I HDACs, HDAC8 directly binds to and delactylates protein arginine methyltransferase 1 (PRMT1) at K134/K145, negatively regulating its methyltransferase activity (Zhou et al., 2025).

The sirtuin family, also known as Class III HDACs, is a highly conserved class of NAD^+^ dependent deacetylases or ADP-ribosyltransferases regulating various cellular processes, including cellular stress response, metabolism, senescence, and apoptosis. This family of seven mammalian members, namely SIRT1 to SIRT7, exhibits distinct functions depending on its subcellular localization and substrate specificity. In general, SIRT1-3 localize in both the nucleus and cytoplasm, SIRT3-5 in the mitochondria, and SIRT6 and SIRT7 only in the nucleus (Chen M. et al., 2024). SIRT1 and SIRT3 are the most extensively characterized delactylases (Du et al., 2024), while SIRT2 and SIRT6 have also been reported to remove lactylation from histone and non-histone substrates (Sun et al., 2023; Zou L. et al., 2025). Overall, delactylation is dynamically controlled by subcellular localization, substrate accessibility, and metabolic state.

##### 2.2.1.6 The “readers”

In contrast to the well-established “writers” and “erasers” of lactylation, the identification of its “readers” remains less well defined. Current evidence suggests that several proteins originally recognized as acetyl-lysine readers may also recognize lactylated lysine residues. The currently identified “reader” proteins mainly include bromodomain-containing proteins (bromodomain-containing protein 2 (BRD2), BRD4, and tripartite motif-containing 33β (TRIM33β)), YEATS domain proteins (AF9, ENL, GAS41, and YEATS2), and plant homeodomain (PHD) finger domain-containing proteins (BPTF, DPF2, and ING) (Sheng et al., 2025) (Table 1, Figure 1). Reported examples include BRG1, TRIM33β, and DPF2, which have been implicated in the interpretation of H3K18la or H3K14la in specific transcriptional settings (Hu et al., 2024; Nuñez et al., 2024; Zhai et al., 2024). However, these “readers” generally exhibit lower affinity for lactylation than acetylation, suggesting the potential existence of specialized or functionally differentiated reading mechanisms to decode lactylation signals precisely (Sheng et al., 2025).

#### 2.2.2 Cross-talk between lactylation and other PTMs

PTMs are chemical modifications of proteins that occur after protein translation and regulate protein activity, stability, localization, and biological function. Common PTMs include acetylation, phosphorylation, ubiquitination, methylation, glycosylation, crotonylation, and succinylation (Zhao L. et al., 2025). Different types of PTMs govern essential cellular processes and contribute to disease pathogenesis. Rather than acting independently, different PTMs can interact synergistically or antagonistically to shape protein function and signaling output (Gong et al., 2024). Lactylation has been reported to interact with several PTMs, particularly acetylation, and phosphorylation (Gong et al., 2024).

The crosstalk between lysine lactylation (Kla) and lysine acetylation (Kac) is especially important because both are metabolically linked lysine modifications whose levels are largely determined by lactate- and acetyl-CoA–dependent metabolism (Zhang et al., 2019; Wellen et al., 2009). Their interplay is further supported by shared enzymatic machinery, as p300/CBP can catalyze both modifications, whereas HDAC/SIRT family members may remove both lactyl and acetyl groups (Zhang et al., 2019; Du et al., 2024; Ogryzko et al., 1996; Zhang Y. et al., 2026). Such enzyme sharing suggests that Kla and Kac may compete at identical or adjacent lysine residues, with the dominant modification depending on substrate concentration, enzyme abundance, catalytic affinity, and local metabolic context (Ma et al., 2025). Therefore, some effects attributed to lactylation may actually reflect shifts in the Kla–Kac balance rather than lactylation alone (Yang K. et al., 2022; Di et al., 2025). Moreover, pharmacological activation or inhibition of shared writers or erasers can simultaneously affect both modifications, further complicating phenotype interpretation (Yang K. et al., 2022). Thus, distinguishing site-specific lactylation from acetylation is essential for defining which PTM truly drives a given biological effect.

Recent investigations have expanded the bidirectional interaction between phosphorylation and lactylation in disease pathogenesis. Phosphorylation signaling pathways, such as PI3K/AKT, mTOR, AMPK, MAPK, NF-κB, Wnt/β-catenin, can promote lactylation via upregulating glycolytic enzymes and lactate production (Xu et al., 2023; De Nardo, 2024; Yang L. et al., 2024; Lu F. et al., 2025). ERK can also directly phosphorylate lactylation enzyme ACSS2, promoting its nuclear translocation and catalyzing the production of lactyl-CoA (Zhu R. et al., 2025). Additionally, AMPK directly phosphorylates p300, promoting its nuclear retention and potentially influencing lactylation dynamics (Son et al., 2024). Conversely, lactylation can modulate the activity of signal transduction networks implicated in cell proliferation, cancer invasion and metastasis, and inflammation (Wang Y. et al., 2024; Zhao et al., 2024b; Zhang et al., 2025a; Zhang et al., 2026b). For example, glycolysis-driven lactate promotes AMPKα lactylation, suppressing its kinase activity and phosphorylation (Zhang et al., 2024). Overall, current findings support the concept that lactylation is integrated into broader PTM networks, although the site-specific competitive or cooperative relationships among different modifications remain insufficiently defined.

## 3 Lactylation in the female reproductive system: molecular mechanisms and clinical implications

Lactylation modification is widely distributed across reproductive tissues and participates in both physiological activities and pathological conditions. In pathological contexts, lactylation modulates disease-relevant processes such as immune dysregulation, mitochondrial redox imbalance, and cell death signaling. These mechanisms are closely associated with inflammation-related reproductive disorders, including polycystic ovary syndrome (PCOS), primary ovarian insufficiency (POI), and endometriosis, as well as gynecological tumor progression, immune escape, and therapeutic resistance. By integrating the latest findings on lactylation in the female reproductive system, this review highlights the pivotal role of lactylation in the development and clinical treatment of female reproductive disease, with specific lactylation sites summarized in Table 2.

**TABLE 2:** Targets and functions of lactylation modifications in the reproductive system.

Disease	Lactylation site	Mechanism	Function	Ref
Oocyte maturation and early embryogenesis	H3K23la, H3K18la	Low O_2_ → Glycolysis↓ → Lactate↓ → Histone lactylation↓	Impair embryonic development to blastocyst	Yang et al. (2021)
Oocyte maturation and early embryogenesis	H3K9la, H3K14la, H4K8la, H4K12la, H4K5la	Exogenous lactate → Histone lactylation↑→ Altered glycolysis pathways	Improve oocyte maturation and embryo development	Yang D. et al. (2024)
Oocyte maturation	H3K18la	Lactate → H3K18la↑ → Meiotic progression	Oocyte maturation and developmental competence	Li D. et al. (2026)
Oocyte maturation	H3K18la, H4K12la	Tfap2a in type 2 diabetes↑→ p300↑ → Histone acetylation & lactylation↑ → Chromosome defects	Hinder mouse oocyte meiosis	Lin et al. (2022)
Granulosa cells	H3K18la; CREB K136la	Hypoxia + hCG → Glycolysis/Lactate↑→ H3K18la, CREB K136la↑ → Steroidogenic genes transcription↑	Enhance granulosa cell luteinization	Wu et al. (2024)
Granulosa cells	CREB K136	FSH →Glycolysis/Lactate↑ → CREB K136la → CREB phosphorylation (S133)↑ & CBP/p300 recruitment → Proliferation and differentiation genes↑	Promote follicular development	Wu G. et al. (2025)
Granulosa cells	H4K5la	FSH → Glycolysis/Lactate↑ → H4K5la↑→ HDAC4↑ → PGC-1α deacetylation at K329/330 → PGC-1α binding to NRF1/NRF2↑ → Mitochondrial biogenesis	Promote proliferation and follicular development	Wu et al. (2026)
Embryonic development	H3K18la	Lactate deficiency → H3K18la ↓ → ZGA gene expression ↓	Failure of ZGA	Zhao et al. (2024a)
Embryonic development	H3K18la	Lactate in nuclear pool↑ → H3K18la↑→ Major ZGA genes	Promote ZGA gene transcription	Li J. et al. (2024)
Embryonic development	H4K12la	Glycolysis↑ in PrE precursors → Lactate↑ → Lactate shuttle to epiblast → FGF4↑→FGFR in PrE precursors → Lactate↑→ H4K12la↑ →PrE genes↑	Promote primitive endoderm (PrE) specification	Hu et al. (2025a)
Embryonic development	H4K12la, H4K8la	AtmosO_2_ → HIF-1α↓→ Glycolysis↓ →H4K12la, H4K8la↓	Impair developmental potential	Yao et al. (2024)
Embryonic development	H4K12la, Pan-Kla	Vitrification → ROS↑ → LDHA/LDHB/p300↓ →H4K12la↓	Impair embryo development	Wei et al. (2024b)
Preeclampsia	HK2-K346la	Oxidative stress → Lactate↓ → HK2-K346la↓ → HK2-VDAC1 binding↓ → Glycolysis	Reduce trophoblast proliferation	Chen Z. et al. (2025)
Preeclampsia	PKM2-K305la	↓HLA-F → PKM2↓ & PKM2-K305la↑ → PKM2 enzyme activity↓ → Glycolysis↓	Impair trophoblast proliferation	Xu R. et al. (2025)
Preeclampsia	H3K18la	Hypoxia → Lactate↑ → H3K18la↑→ FN1 & SERPINE1 expression↑	Placental fibrosis	Li et al. (2022)
Preeclampsia	H3K18la	Lactate↑ → H3K18la↑ →GADD45A↑ → P16↑	Trophoblast senescence	Li X. et al. (2025)
Preeclampsia	BCAT2 K377la	Hypoxia → Lactate↑ →BCAT2 lactylation↑ → BCAT2 ubiquitination & degradation↑	Promote trophoblast dysfunction and oxidative stress	Yin et al. (2026)
Preeclampsia	Hsp60-K469la; K473la	Hypoxia → Lactate↑ → Hsp60 K469/K473 lactylation↑→ Drp1-Ser616 phosphorylation↑ → Mitochondrial apoptosis	Trophoblast apoptosis and invasion defect	Xu J. et al. (2025)
Gestational diabetes mellitus	H3K18la	Lactate↑ → H3K18la↑ → CACNA2D1↑	Promote trophoblast viability and proliferation	Huang X. et al. (2024)
Endometrial receptivity	H4K12la	Progesterone → Glycolysis/lactate↑ → H4K12la → HIF-1α–glycolysis feedback	Decidualization	Zhao et al. (2023)
Endometrial receptivity	H3K18la	Conceptus-derived lactate↓ → H3K18la↓ → Unbalanced redox homeostasis and apoptosis	Impair implantation	Yang Q. et al. (2022)
Endometrial receptivity	H3K18la	LDHA↓ → Lactate↓ → H3K18la↓ → SLC7A11↓ →EMT & Cell migration↓	Impair endometrial receptivity	Dong et al. (2025)
Endometrial receptivity	H3K18la	PCOS → Endometrial ERα↑ → LDHA/LDHB↑ → Lactate↑ → H3K18la↑ → Estrogen-responsive genes↑	Reduce implantation rate and live birth rate	Shan et al. (2026)
Endometriosis	H3K18la	lncRNA H19↑ → Glycolysis/lactate↑ → H3K18la↑	Increase proliferation and migration	Wen et al. (2024)
Endometriosis	H3K18la	Lactate↑ →H3K18la↑ → HMGB1↑ → PI3K/AKT pathway	Increase proliferation, migration, and invasion	Chen et al. (2023)
Endometriosis	H3K18la	H3K18la↑ → RASD2↑ → CTPS1 SUMOylation↑ & Ubiquitination↓, protein stability↑→ Nucleotide biosynthesis	Increase proliferation, migration, and invasion	Wang Z. et al. (2025)
Endometriosis	H3K18la	Lactate↑ → H3K18la↑ → METTL3↑ →m^6^A modification↑+ HIF1A transcription↑→ HMOX1↑→ GPX4↑& ACSL4↓	Ferroptosis resistance, survive under oxidative stress	Liang et al. (2025)
Endometriosis	Snail1 lactylation	AARS1→ Snail1 lactylation↑ → Ubiquitination↓ →Snail1 stability↑	Promote proliferation, migration, invasion, and EMT	Liu L. et al. (2025)
Polycystic ovary syndrome	H3K9la, H3K18la, H3K27la	Androgen → PKM2 nuclear accumulation →H3K9/18/27la↑ → 3D genome remodeling → PCOS gene activation	Upregulate PCOS-related genes	Yu et al. (2025a)
Polycystic ovary syndrome	PRDX6 K209la	SESN2↓ → PRDX6 K209la↓ →PRDX6-GPX4 interaction↓ → Ferroptosis↑	Ovarian dysfunction	Li Y. Y. et al. (2026)
Premature ovarian insufficiency	CPT2 K457/8la; PDHA1 K336la	AARS2↑ → CPT2 & PDHA1 Lactylation↑ & activity↓ → PPARγ & mTORC1 signaling↑→ Granulosa cell proliferation↑, FSH sensitivity↑	Premature follicle depletion	Zhang Z. L. et al. (2025)
Premature ovarian insufficiency	CPT2 K457/8la; PDHA1 K336la	AARS2 R194C/R199C → PDHA1/CPT2 lactylation↑ & activity↓→ mTORC1 activity↑→ Granulosa cell proliferation↑	Primordial follicle activation	Zhang H. H. et al. (2026)
Ovarian cancer	PFKP K392la	Hypoxia → PFKP K392 lactylation & stabilization → PTEN↓ → Glycolysis↑	Promote tumor growth	Mi et al. (2025)
Ovarian cancer	MRE11/NBS1	Cisplatin → Pyruvate↓→ ACAT1 binds ME2↑→ ME2 K156 acetylation↑ & activity↑ → Conversion of malate to pyruvate/lactate↑ → Lactylation of HR proteins↑	Promote DNA repair and platinum resistance	Zheng et al. (2025)
Ovarian cancer	H4K12la	LDHC4↑ → Lactate↑ → H4K12la↑ at PGK1 promoter → Glycolysis↑	Promote proliferation, migration, invasion, metastasis	Lin et al. (2026)
Ovarian cancer	H3K18la	Tanshinone I → FOXK1/2↓ → Glycolysis/Lactate↓ → H3K18la↓ →oncogenic targets↓	Inhibit tumor growth and alleviate immunosuppressive TME	Jin et al. (2025)
Ovarian cancer	H3K9la; RAD51 K73la	Lactate↑→ GCN5-mediated H3K9la & RAD51 K73la → HR repair↑	Platinum (Cisplatin) resistance	Sun et al. (2025)
Ovarian cancer	H4K12la	Glycolysis/Lactate↑→ H4K12la↑ → MYC recruitment → RAD23A↑ → DNA repair↑	PARP (Niraparib) resistance	Lu B. et al. (2025)
Ovarian cancer	H3K18la	Glycolysis/Lactate↑ → H3K18la↑ → TRA2A↑ → Ferroptosis↓	Platinum (Cisplatin) resistance	Gao M. et al. (2025)
Ovarian cancer	H3K18la	Tumor lactate → binds GPR132 on macrophages → H3K18la↑ → CCL18↑ → M2 polarization	Promote metastasis, invasion	Sun J. et al. (2024)
Ovarian cancer	H3K18la	LDHB↑ → Lactate↑ → H3K18la↑ → PD-L1↑	Promote immune escape	Hu et al. (2025b)
Ovarian cancer	Global histone lactylation, TOX	FOXK1↑→ Glycolysis/Lactate↑ → Histone lactylation↑ → TOX lactylation↑ → CD8^+^ T cell exhaustion	Promote immune evasion, tumor progression	Li (2026)
Cervical cancer	DCBLD1 K172la	Lactate↑ → HIF-1α activation↑ → DCBLD1↑ & K172 lactylation↑ → G6PD stabilization↑ →PPP↑	Promote proliferation and tumor growth	Meng et al. (2024a)
Cervical cancer	G6PD K45la	HPV16 E6 → LDHA↓ → Lactate↓ → G6PD K45la↓ → Enzymatic activity↑ → PPP↑	Promote cell proliferation	Meng et al. (2024b)
Cervical cancer	PPP1R14B K140la	PPP1R14B K140la↓→ Prognostic subtypes	Suppress proliferation and migration	He et al. (2024)
Cervical cancer	H3K14la	H3K14la recognition by DPF2 → Activation of oncogenic programs	Enhance proliferation and survival	Zhai et al. (2024)
Cervical cancer	H3K18la	Tumor lactate↑ → Macrophage H3K18la↑ →GPD2↑ → M2 polarization	Immune evasion	Huang C. et al. (2024)
Cervical cancer	H3K18la	ICAT↑ → Glycolysis/lactate↑ → H3K18la↑ in TAMs→ M2 polarization	Immune evasion	Dang et al. (2025)
Endometrial cancer	H3K18la	Lactate↑ → H3K18la↑ → USP39↑→ PGK1 stabilization↑ → PI3K/AKT/HIF-1α↑ → Glycolysis↑	Promote tumor progression	Wei S. et al. (2024)
Endometrial cancer	PFKM K678la	PFKM K678 lactylation↑ → PFKM activity↑ → Glycolysis/Lactate↑	Promote proliferation, invasion, and angiogenesis	Wang B. et al. (2025)
Endometrial cancer	H3K18la	Hypoxia → Glycolysis/Lactate↑ → H3K18la↑→ NHE7↑ → ER stress↑	Promote progression	Yang et al. (2026a)
Endometrial cancer	H3K18la	Tumor-derived lactate↑→ Macrophage MCT1 uptake↑→ H3K18la↑→ DNMT1↑ → NHE7 silencing via methylation → M2 polarization	Promote proliferation, migration, and invasion	Yang et al. (2026b)

### 3.1 Lactylation in reproduction and early embryonic development

#### 3.1.1 Lactylation and oocyte maturation

In mammals, oogonia enter meiosis during fetal life and arrest at prophase I as germinal vesicle (GV) oocytes. After puberty, hormonal stimulation triggers meiotic resumption through germinal vesicle breakdown (GVBD), followed by progression to metaphase I (MI) and arrest at metaphase II (MII) until fertilization. This extended progression depends on the precise coordination of transcriptional and epigenetic regulation. Histone lactylation regulates chromatin state, thereby functioning as a key mediator of oocyte quality and maturation (Eckersley-Maslin et al., 2018). During oogenesis, histone lactylation displays stage- and site-specific patterns that are closely linked to functional transitions. Histone lactylation marks are highly abundant at the GV stage and decline following meiotic resumption (Yang D. et al., 2024; Yang et al., 2021). Global Pan-Kla levels decrease at MII compared with the GV stage, and selective marks such as H3K23la persist on condensed chromosomes (Yang et al., 2021). Detailed profiling reveals enrichment of H3K9la, H3K14la, H4K8la, and H4K12la at GV, with a transient peak of H4K5la at GVBD (Yang D. et al., 2024).

Functional studies further support the importance of balanced lactate-lactylation signaling in oocyte development. Exogenous sodium lactate increases histone lactylation, improves oocyte maturation, and alters the expression of genes related to oxidative phosphorylation and meiosis (Yang D. et al., 2024). Conversely, inhibition of lactate metabolism in goat oocytes reduces H3K18la, leading to mitochondrial dysfunction and disrupted meiosis (Li D. et al., 2026). However, lactylation is not simply beneficial. In a type 2 diabetes mouse model, Tfap2a-driven upregulation of p300 causes excessive histone acetylation and lactylation, disrupting spindle assembly and chromosome alignment and ultimately causing meiotic failure (Lin et al., 2022). These findings suggest that maintaining appropriate levels of lactate and lactylation is important for oocyte development.

#### 3.1.2 Lactylation and granulosa cells

In female reproduction, oocytes and granulosa cells (GCs) form an integrated metabolic unit. Granulosa cells are essential for follicular activation, oocyte growth, and hormone production (Zhang and Liu, 2015). Recent studies have identified that lactylation is an important regulatory layer in both GC metabolism and hormone-driven differentiation. Comprehensive lactylome analysis of porcine GCs revealed over 24,000 lactylated sites across metabolic pathways (Fan et al., 2025). Under hypoxic follicular conditions, human chorionic gonadotropin (hCG) induces lactate production and promotes H3K18 and cAMP response element-binding protein (CREB) K136 lactylation, thereby activating steroidogenic genes CYP11A1 and STAR to promote progesterone synthesis and luteinization (Wu et al., 2024). Similarly, follicle-stimulating hormone (FSH) drives glycolysis and p300-dependent CREB lactylation at K136. This modification enhances CREB phosphorylation while recruiting the CBP/p300 coactivator complex to drive steroidogenesis and differentiation (Wu G. et al., 2025). FSH-induced lactate also drives p300/CBP-dependent H4K5 lactylation at the HDAC4 promoter. Such lactylation activates HDAC4, which deacetylates peroxisome proliferator-activated receptor gamma coactivator 1-alpha (PGC-1α) at sites K329/330, enhancing its binding capacity to the nuclear respiratory factor (NRF) 1/2, thereby resulting in an increase in mitochondrial biogenesis. This supports GC proliferation and estradiol synthesis, thus sustaining normal follicular development (Wu et al., 2026). Taken together, lactylation translates gonadotropin stimulation into metabolic and transcriptional competence.

#### 3.1.3 Lactylation and embryonic development

Preimplantation embryonic development spans from fertilization to blastocyst implantation into the uterine endometrium around day 7. This critical window is characterized by zygote genome activation, successive cleavage divisions, morula formation, and ultimately blastocyst hatching and invasion. Following implantation, the mammalian embryo undergoes gastrulation to establish the three primary germ layers (ectoderm, mesoderm, and endoderm), which is followed by early organogenesis (Zhai et al., 2022).

During early embryonic development in mammals, the metabolic pattern undergoes significant changes, transitioning from an oxidation-phosphorylation-dominated regime to glycolysis-dominated dominance and ultimately to mixed metabolism, while relying on nutrients provided by the maternal environment; lactate serves as one of the primary substrates for embryonic energy metabolism. Maternal-derived LDHB is crucial for maintaining redox balance and developmental potential during the early stages of fertilized egg division (Deng et al., 2026). During early mammalian embryogenesis, as development progresses to the blastocyst stage, lactate production further increases. This metabolic characteristic closely coincides with the second cell fate determination event when the inner cell mass differentiates into the epiblast and primitive endoderm (Hu et al., 2025a). The rapid cell proliferation of blastocyst cells requires aerobic glycolysis to support biosynthesis and redox homeostasis, which resembles the Warburg effect of cancer cells (Krisher and Prather, 2012). For example, during the early stage of neural tube closure in mouse embryos, physiological hypoxia induces the expression of glycolytic genes in neuroepithelial cells; inhibition of glycolysis leads to defects in neural tube closure, thereby confirming the necessity of glycolytic activity during specific developmental windows (Sakai et al., 2023). Furthermore, the Notch signaling pathway mediates glycolytic switching via the PI3K/AKT signaling cascade to support the escalating energy demands during hypoxic conditions from mid to late embryonic development (Wang H. et al., 2023). These dynamic metabolic changes provide an ample substrate foundation for lactylation modification, enabling the metabolic state to be directly translated into epigenetic signals.

In line with cellular demands for glycolysis and lactic acid production, lactylation exhibits significant spatiotemporal dynamic variations during early embryonic development in mammals. After fertilization, the lactylation signal significantly weakened in the zygote but was uniformly distributed in the paternal and maternal pronuclei, suggesting its possible involvement in the symmetrical resetting of the parental genome epigenetic state. During the cleavage stage of the embryo (e.g., from the 2-cell to the morula stage), the level of lactylation still remained low, but significantly increased during the blastocyst stage (H4K5la and H4K12la), possibly reflecting the demand for metabolic reprogramming during embryonic genome activation and trophoblast differentiation (Yang D. et al., 2024). In another study, immunofluorescence staining analysis also demonstrated that H3K23la and H3K18la are weakly present in mouse zygotes, but increase during cleavage, and peak at the blastocyst (Yang et al., 2021).

During early embryonic development, a key event is zygotic genome activation (ZGA), which marks the transition of regulatory control from maternal control to embryonic transcription (Schul et al., 2019). ZGA is closely linked to embryonic metabolic states and is under epigenetic control (Chen Y. et al., 2020). In mouse and human embryos, nuclear lactate accumulates around the major ZGA stage, and nuclear lactate promotes H3K18la enrichment at ZGA gene promoters. Experiments involving LDH inhibition, H3K18R mutation, and Lac-CoA rescue confirmed that this mechanism is conserved in human embryos (Li J. et al., 2024). Another study similarly showed that lactate deficiency impairs maternal-to-zygotic transition and reduces H3K18la, whereas H3K27ac shows limited association with the transcriptional defects (Zhao et al., 2024a).

Lactylation plays active and stage-specific roles in cell fate determination. During the second cell fate decision, lactate production increases preferentially in primitive endoderm precursor cells. Lactate and epiblast-derived FGF signaling reciprocally activate each other and form an intercellular positive feedback loop that promotes primitive endoderm formation (Hu et al., 2025a). Consistently, adding 10 mM sodium lactate to the culture medium enhances embryo cleavage and blastocyst formation rates, supporting a developmental role for properly regulated lactylation (Yang D. et al., 2024).

Embryonic development appears highly sensitive to lactylation dosage, as both insufficient and excessive lactylation are detrimental. Culture conditions can reshape embryonic lactylation and developmental competence. Hypoxic *in vitro* culture at 2% O_2_ reduces H3K23la, H3K18la, and pan-histone lactylation and impairs embryonic developmental potential (Yang et al., 2021). Similarly, in preimplantation mouse embryos, exposure to atmospheric oxygen (20% O_2_) significantly reduces H4K12la and H4K8la in blastocysts, which is associated with impaired developmental potential (Yao et al., 2024). These findings indicate that appropriate oxygen and lactate metabolism are required to maintain embryonic lactylation homeostasis.

Stress-related embryo models further show that both insufficient and excessive lactylation may impair embryo development. In embryos derived from vitrified MII oocytes, oxidative stress downregulates LDHA, LDHB, and p300, leading to reduced H4K12la levels and developmental arrest. This defect can be rescued by the antioxidant peptide TW-7, which restores histone lactylation (Wei et al., 2024b). In aged embryos, however, calcium dyshomeostasis promotes LDHA upregulation and increases lactate production and global protein lactylation, which compromises embryo development competence. This defect can be partially rescued by amphiregulin through restraining excessive LDHA-driven lactylation (Yang D. et al., 2025).

Lactylation has practical implications for assisted reproduction. Embryo culture, oocyte *in vitro* maturation, sperm preparation, and follicle culture all expose cells to artificial nutrient and oxygen conditions. The observation that both insufficient and excessive lactate/lactylation can impair embryo competence suggests that culture systems should be optimized not only for blastocyst rate, but also for stage-specific epigenetic fidelity. Lactylation markers such as H3K18la, H4K12la, and selected non-histone substrates may eventually serve as quality-control readouts in experimental reproductive technologies. Lactylation-targeted interventions in fertility medicine require precise dosing and timing.

### 3.2 Lactylation in pregnancy diseases

#### 3.2.1 Lactylation and preeclampsia

Preeclampsia (PE), a pregnancy-specific hypertensive disorder caused by placental dysfunction, is one of the main causes of maternal and perinatal morbidity and mortality globally. It is characterized by impaired trophoblast invasion, inadequate spiral artery remodeling, and subsequent placental hypoxia and oxidative stress (Rana et al., 2019). These placental abnormalities lead to the release of antiangiogenic factors, inflammatory mediators, and metabolic stress signals into the maternal bloodstream, triggering systemic endothelial dysfunction and preeclampsia (Rana et al., 2019). Recent lactylome studies show that placental lactylation is globally altered in PE, with related pathways involving vascular smooth muscle contraction, platelet activation, and cholesterol metabolism. These findings suggest that lactylation may connect placental metabolic dysfunction with vascular and endothelial abnormalities in PE (Feng et al., 2025).

Non-histone lactylation regulates glycolytic enzymes in trophoblast and endothelium in a site-specific manner. In placental vascular endothelial cells, hypoxia-induced upregulation of HK2 enhances glycolysis and lactate production, which drives global protein lactylation and activates NLR family pyrin domain-containing 3 (NLRP3)-mediated pyroptosis, consequently exacerbating endothelial injury (Lu X. et al., 2025). In trophoblasts, by contrast, oxidative stress impairs HK2 K346 lactylation, which disrupts its interaction with voltage-dependent anion channel 1 (VDAC1) and impairs mitochondrial localization. This loss of HK2 lactylation decreases hexokinase activity and ultimately compromises trophoblast proliferation (Chen Z. et al., 2025). As a key rate-limiting enzyme in glycolysis, HK2 exhibits significant variations in its subcellular localization and metabolic flux regulation across different cell types, resulting in distinct spatial distributions of lactate production and lactate substrates. Beyond catalyzing glucose conversion to glucose-6-phosphate, HK2 also couples glycolysis with oxidative phosphorylation by binding to the VDAC in the mitochondrial outer membrane, thereby regulating the intracellular ATP/ADP ratio and lactate generation rate (Peng and Du, 2025). HK2 exhibits distinct responses to lactylation modification and different regulatory roles in disease progression between placental vascular endothelial cells and trophoblasts during preeclampsia, a phenomenon that profoundly reflects the specificity of various cell types in metabolic reprogramming, epigenetic regulation networks, and microenvironmental response mechanisms. Furthermore, cell-specific gene regulatory networks exhibit fundamental differences in their response to lactylation modifications. As a mechanism that couples metabolic states with gene expression, the ultimate biological effects of lactylation modifications depend on the function of downstream target genes (Merkuri et al., 2024). In trophoblast cells, lactylation modifications may predominantly accumulate at gene loci regulating cellular proliferation, migration, and invasive capacity, such as VEGF and HIF-1α, to meet the demands of placental implantation and spiral artery remodeling (Malkowska et al., 2022). In vascular endothelial cells, lactylation modification may predominantly regulate genes related to vascular permeability, coagulation function, and inflammatory responses. The core pathological features of preeclampsia include systemic vascular endothelial dysfunction and inadequate placental perfusion; the functional imbalance between these two cell types collectively contributes to disease pathogenesis.

In PE placentas, human leukocyte antigen F (HLA-F) expression is reduced in extravillous trophoblasts and villous cytotrophoblasts. HLA-F promotes trophoblast proliferation by increasing PKM2 transcription and protein expression. It also enhances PKM2 activity by reducing PKM2 K305 lactylation. Loss of HLA-F therefore suppresses PKM2-dependent glycolysis and trophoblast proliferation (Xu R. et al., 2025).

Histone lactylation further converts the metabolic stress of ischemic and hypoxic conditions into pathogenic transcriptional programs. PE placentas show increased lactate, global lactylation, and H3K18la levels. In trophoblast cell lines, hypoxia increases lactate production and H3K18la, whereas oxamate reduces these effects. Mechanistically, H3K18la is enriched at the promoters of FN1 and SERPINE1, thereby promoting the expression of fibrosis-related genes (Li et al., 2022). Lactate-driven histone lactylation also contributes to premature placental senescence. Lactate induces H3K18la enrichment at the promoter of growth arrest and DNA damage-inducible alpha (GADD45A), which transcriptionally upregulates GADD45A to drive premature placental senescence (Li X. et al., 2025).

Mitochondrial non-histone lactylation has emerged as a pathogenic mechanism that links hypoxia-driven lactate accumulation to redox imbalance, mitochondrial dysfunction, and impaired trophoblast function in preeclampsia. Yin et al. demonstrated that hypoxia-induced lactate drives p300-mediated lactylation of branched chain amino acid transaminase 2 (BCAT2) at K377 in trophoblasts. This modification does not alter BCAT2 enzyme activity but promotes BCAT2 ubiquitination and degradation. Given the mitochondrial localization of BCAT2, its lactylation further disturbs redox homeostasis and increases reactive oxygen species (ROS) production. As a result, trophoblast migration, invasion, and tube formation decline, while oxidative stress increases (Yin et al., 2026). Heat shock protein 60 (Hsp60) is also aberrantly lactylated in PE trophoblasts. Hsp60 lactylation at K469 and K473 promotes dynamin-related protein 1 (Drp1) phosphorylation at Ser616. This triggers excessive mitochondrial fission, ROS generation, and cytochrome c release, ultimately activating caspase-3-dependent apoptosis and impairing trophoblast invasive capacity (Xu J. et al., 2025).

**FIGURE 2:**
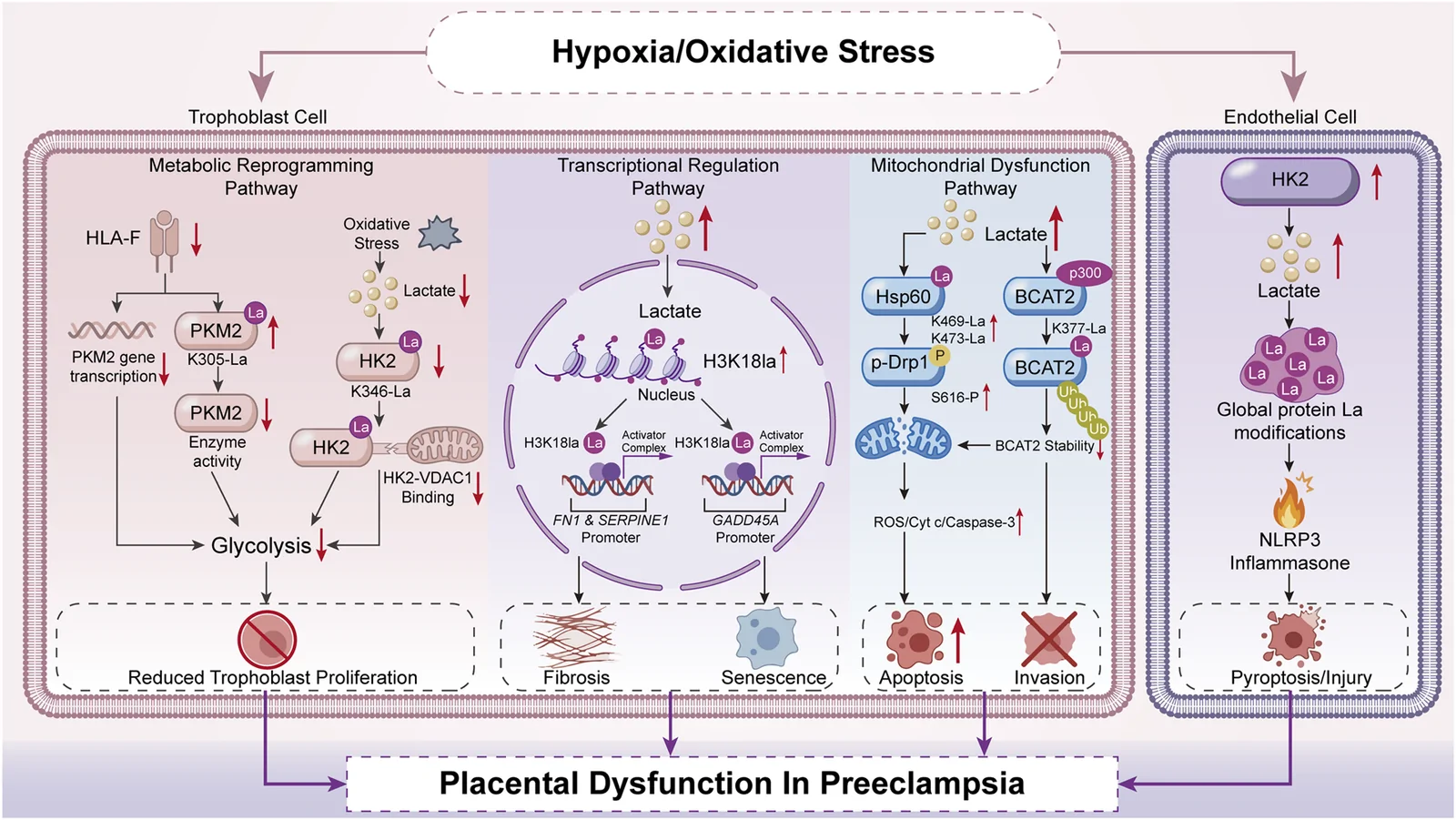
Lactylation-mediated placental dysfunction in preeclampsia. Hypoxia- and oxidative stress-associated metabolic reprogramming may alter histone and non-histone lactylation in placental cells. Dysregulated lactylation is implicated in trophoblast dysfunction, endothelial injury, inflammatory activation, impaired placental homeostasis, and the development of preeclampsia.

Collectively, lactylation participates in PE through multiple placental compartments and molecular layers. These mechanisms converge on impaired trophoblast proliferation and invasion, mitochondrial dysfunction, endothelial pyroptosis, oxidative stress, and placental vascular disorder. Targeting lactate production, lactylation writers or erasers, and site-specific lactylation events may provide new strategies for understanding and managing PE (Figure 2).

#### 3.2.2 Lactylation and gestational diabetes mellitus

Gestational diabetes mellitus (GDM) refers to impaired glucose intolerance that first appears during pregnancy and is one of the most common metabolic complications of pregnancy. GDM increases the risk of adverse maternal, fetal, and long-term metabolic outcomes, including preeclampsia, risk of developing type 2 diabetes, macrosomia, and neonatal metabolic disturbances (McIntyre et al., 2019; Sweeting et al., 2022). It arises from pregnancy-induced insulin resistance combined with inadequate pancreatic β-cell compensation, leading to maternal hyperglycemia and altered placental glucose metabolism (Sweeting et al., 2022). At the maternal-fetal interface, this metabolic state enhances glycolysis and lactate production, which in turn promotes H3K18 lactylation and upregulates target genes, including CACNA2D1, thereby supporting trophoblast viability and proliferation. Notably, seven lactylation-responsive genes were associated with glucose metabolism and insulin regulation, suggesting that lactate-driven histone lactylation modulates trophoblast function in GDM (Huang X. et al., 2024). However, evidence directly linking lactylation to GDM remains limited. The cell-type-specific lactylation patterns at the maternal-fetal interface, and their potential impact on insulin signaling, placental nutrient transport, and fetal metabolic programming remain largely unexplored.

#### 3.2.3 Lactylation and endometrial receptivity

Endometrial receptivity is the ability of the endometrium to allow the embryo to successfully adhere, invade, and complete implantation. This process is highly time- and space-specific and is precisely regulated by hormones, cytokines, adhesion molecules, and epigenetic modifications. At the cellular level, receptivity manifests as changes in epithelial polarity, pinopode formation, and stromal decidualization (Neykova et al., 2022; Pathare et al., 2023). Impairment of endometrial receptivity is closely associated with negative reproductive outcomes, notably recurrent implantation failure (RIF) and recurrent pregnancy loss (RPL) (Pathare et al., 2023).

During normal decidualization, glycolysis and lactate synthesis are significantly upregulated to meet the demands of invasion and placental development, resembling a Warburg-like metabolic state (Zuo et al., 2015). The Warburg effect promotes endometrial receptivity by establishing a high-lactate and low-pH microenvironment, which induces histone lactylation and glycolytic reprogramming, thereby facilitating decidualization and embryo implantation (Zhang X. et al., 2025). MCT4-positive decidual cells can export lactate, whereas adjacent MCT1-positive stromal cells import it to sustain proliferation, whereas pharmacological blocking of glycolysis or lactate transport compromises decidual development (Zuo et al., 2015). Lactate exchange at the placental interface also helps maintain early pregnancy. Cytotrophoblast-derived lactate is transported to syncytiotrophoblasts via MCT1, where it activates the PI3K–AKT–mTOR–SREBP1 pathway and upregulates stearoyl-CoA desaturase 1 (SCD1) and glutathione peroxidase 4 (GPX4), thereby limiting lipid peroxidation and ferroptosis (Zhu Y. et al., 2025). In contrast, RPL exhibit reduced glycolysis, impaired lactate synthesis and transport, and elevated ferroptosis markers, which are associated with trophoblast dysfunction, including impaired proliferation and increased apoptosis (Zhu Y. et al., 2025; Zhu et al., 2023; Gou et al., 2025).

**FIGURE 3:**
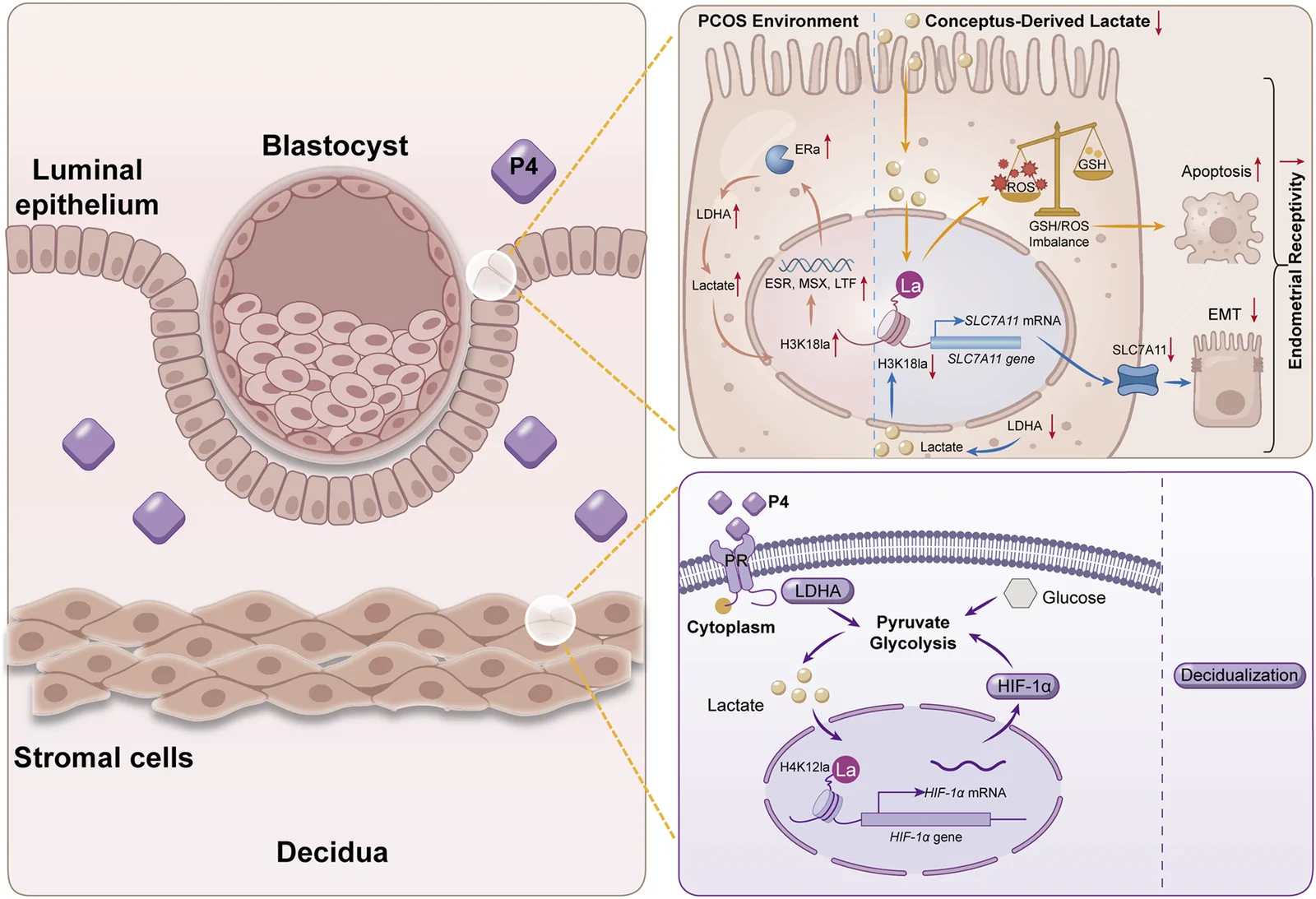
Lactylation in endometrial receptivity and decidualization. Lactate-dependent histone lactylation links metabolic remodeling to epithelial and stromal cell function in the endometrium. Physiological lactylation supports decidualization and implantation, whereas dysregulated lactate–lactylation signaling may disrupt redox balance, epithelial plasticity, and endometrial receptivity.

Lactate-induced lactylation directly regulates gene expression and cellular function at the maternal-fetal interface. Progesterone produced during pregnancy directly upregulates LDHA expression, which increases lactate production and drives H4K12 lactylation. H4K12la then activates HIF-1α expression and glycolysis, forming a positive feedback loop that promotes endometrial stromal cell decidualization (Zhao et al., 2023). In sheep, conceptus-derived lactate similarly enhances endometrial preparation through H3K18la-dependent regulation of redox balance. This effect is concentration-dependent, as both lactate deficiency and excess are detrimental to implantation (Yang Q. et al., 2022) (Figure 3).

In RIF, impaired endometrial receptivity may reflect insufficient lactate-dependent epigenetic activation. Dong et al. found reduced LDHA expression in RIF endometrium and showed that lactate enhances H3K18la in endometrial epithelial cells. H3K18la enriches at the SLC7A11 promoter and upregulates SLC7A11, which promotes EMT, cell migration, blastoid adhesion, and blastoid expansion in endometrial organoid and blastoid–endometrial cell implantation models (Dong et al., 2025) (Figure 3).

However, excessive lactylation can also impair implantation. In women with PCOS and in PCOS mouse models, reduced endometrial receptivity is accompanied by elevated ERα expression and increased histone lactylation. ERα increases H3K18la through LDHA/LDHB, while H3K18la further activates estrogen-responsive genes, forming a feed-forward loop. Inhibition of ERα or lactate production reduces H3K18la, restores uterine receptivity, and improves implantation in PCOS mice (Shan et al., 2026) (Figure 3).

In summary, these findings support a context-dependent role of lactate and lactylation in pregnancy loss. Physiological lactylation promotes decidualization, uterine receptivity, and embryo implantation. In contrast, dysregulated lactylation may contribute to RIF and PCOS-associated implantation failure. Future studies should define the site-specific lactylation targets that distinguish protective uterine remodeling from pathological implantation failure.

### 3.3 Lactylation and endometriosis

Endometriosis (EMS) is a prevalent estrogen-dependent chronic inflammatory condition characterized by the ectopic growth of endometrial-like tissue (Vercellini et al., 2014). It impacts approximately 6%–10% of reproductive-aged women, and its prevalence increases to 30%–50% among women with infertility (Ladanyi et al., 2019). Clinically, EMS manifests with chronic pelvic pain, dysmenorrhea, and infertility, which seriously impair patients’ mental health and overall quality of life, creating a considerable medical burden (Vercellini et al., 2014; Taylor et al., 2021). Moreover, a diagnosis of EMS is accompanied by an approximately 50% greater risk of ovarian cancer (Vercellini et al., 2014). Similar to tumors, ectopic lesions exhibit pronounced metabolic reprogramming, particularly enhanced aerobic glycolysis (Kasvandik et al., 2016). This metabolic reprogramming promotes lactate accumulation and facilitates lactylation-mediated epigenetic remodeling.

Histone lactylation, especially H3K18 lactylation, is a key mediator connecting metabolic reprogramming to EMS progression. The upregulation of the long noncoding RNA H19 enhances glycolytic flux by increasing the expression of glycolytic enzymes such as LDHA, PKM2, and ALDOA. Enhanced glycolysis promotes lactate production and global histone lactylation, including H3K18la, thereby promoting stromal cell proliferation and migration. Consistently, sodium lactate enhances histone lactylation, proliferation, and migration, whereas 2-deoxy-D-glucose (2-DG) suppresses these effects (Wen et al., 2024). H3K18la also activates pathogenic transcriptional programs. For example, H3K18la promotes high-mobility group box 1 (HMGB1) expression, which further drives proliferation, invasion, and metabolic support for ectopic lesion growth (Chen et al., 2023). Another H3K18la-dependent pathway involves Ras homolog enriched in striatum 2 (RASD2). RASD2 interacts with CTP synthase 1 (CTPS1) and stabilizes it by promoting SUMO2/3 modification at K38, K171, and K584 while reducing ubiquitination (Wang Z. et al., 2025). Thus, histone lactylation can cooperate with other PTMs to sustain endometriotic cell growth and invasiveness.

Histone lactylation also mediates resistance to regulated cell death. In ectopic endometrial stromal cells, glycolysis-driven lactate accumulation increases H3K18la enrichment at the METTL3 promoter, thereby upregulating METTL3 and activating the m^6^A-dependent HIF1A/HMOX1 pathway to suppress ferroptosis. Consistently, combined treatment with 2-DG and the ferroptosis inducer erastin reduced ectopic lesion volume more effectively than either treatment alone (Liang et al., 2025).

The lactate-sensing enzyme AARS1 is upregulated in ectopic endometrial tissues and promotes proliferation, migration, invasion, and EMT in endometriotic stromal cells. AARS1 catalyzes the lactylation of the EMT-associated transcription factor Snail1, thereby inhibiting Snail1 ubiquitination and degradation and increasing its protein stability. As Snail1 is a central EMT regulator, the AARS1–Snail1 lactylation pathway provides a non-histone mechanism by which lactylation enhances EMT and invasive behavior in EMS (Liu L. et al., 2025).

Therefore, lactylation connects glycolytic reprogramming with epigenetic activation, PTM crosstalk, protein stabilization, ferroptosis resistance, and invasive behavior in EMS. Targeting lactylation-related signaling may help identify diagnostic biomarkers and therapeutic targets, offering new strategies to limit lesion progression and manage EMS-associated infertility.

### 3.4 Lactylation and reproductive endocrine diseases

Reproductive endocrine diseases are characterized by abnormalities in the hypothalamic-pituitary-gonadal axis, reproductive hormones, or their receptors. These abnormalities are frequently associated with systemic metabolic disturbances, forming a reproductive endocrine-metabolic syndrome. Clinical manifestations of these disorders include menstrual irregularities, infertility, abnormalities in sexual development, and metabolic syndrome (McCoskey and Vernon, 2024; Selander-Han et al., 2024).

#### 3.4.1 PCOS

PCOS is a highly prevalent endocrine disorder of the female reproductive system, marked by anovulation, hyperandrogenism, and polycystic ovarian changes, affecting 11%–13% of women worldwide (Stener-Victo et al., 2024). It is one of the primary causes of anovulatory infertility in reproductive-aged women (Escobar-Morreale, 2018). In addition to abnormal manifestations in the reproductive system, it induces systemic metabolic disturbances, including obesity, hyperinsulinemia, dyslipidemia, and chronic low-grade inflammation, which may impair organs beyond the reproductive system, such as the cardiovascular system, liver, and brain, seriously increasing long-term health risks (Zhang et al., 2025b).

GCs are essential for follicular development and oocyte support, and their metabolic dysfunction is closely linked to follicular arrest in PCOS. During normal follicular folliculogenesis, maturing cumulus–oocyte complexes exhibit high glucose and pyruvate consumption to support oocyte competence (Warzych and Lipinska, 2020). In PCOS, however, GCs exhibit reduced glucose uptake and a metabolic shift toward lactate production. This change deprives the oocyte of a critical energy source and ultimately compromises oocyte quality and developmental potential (Maruthini et al., 2014; Liu et al., 2020). Recent studies further link GC metabolic abnormality to altered lactylation. Granulosa cells from women with PCOS exhibit widespread changes in protein lactylation, with significant enrichment in the TGF-β/Smad pathway (Liu et al., 2026).

Histone lactylation links hyperandrogenism to transcriptional dysregulation in PCOS through a PKM2-dependent pathway. In granulosa cells from patients with PCOS, PKM2 is upregulated. Androgen stimulation further promotes PKM2 nuclear translocation through ERK1/2 signaling. Nuclear PKM2 increases nuclear lactate levels and enhances H3K9/H3K18 lactylation, which remodels three-dimensional chromatin architecture and activates steroidogenic genes such as CYP17A1 and CYP11A1. CYP17A1-driven androgen production then forms an androgen–PKM2–histone lactylation feedback loop that reinforces the PCOS phenotype (Yu et al., 2025a).

The pathogenesis of PCOS is associated with ferroptosis, a regulated cell death pathway executed through iron-dependent lipid peroxidation, which promotes granulosa cell dysfunction, follicular atresia, and ovulatory defects (Li X. et al., 2024). Sestrin 2 (SESN2), a stress-inducible metabolic regulator, protects GCs from oxidative damage and ferroptosis under PCOS-related stress. Peroxiredoxin 6 (PRDX6) is a bifunctional antioxidant enzyme that reduces phospholipid hydroperoxides (Lee et al., 2012; Fujita et al., 2024). Under PCOS conditions, SESN2 protects ovarian granulosa cells from ferroptosis by acting as a stress-responsive protective molecule (Li Y. Y. et al., 2026). SESN2 deficiency reduces lactylation of PRDX6 at K209, which weakens the PRDX6–GPX4 interaction and impairs antioxidant defense, thereby sensitizing granulosa cells to ferroptotic injury (Li Y. Y. et al., 2026).

Overall, current studies suggest that lactylation participates in PCOS through both pathogenic and protective mechanisms. However, studies on lactylation in PCOS remain limited, and its roles in PCOS-related inflammation, glucose and lipid metabolic disorders, miscarriage susceptibility, and other clinical complications require further investigation.

#### 3.4.2 POI

Declining ovarian function is a major reproductive endocrine contributor to female infertility. POI is defined as the decline of ovarian function before 40 years of age, and is characterized by elevated FSH levels and markedly reduced ovarian hormone production (Stuenkel and Gompel, 2023). Meta-analyses have estimated the prevalence of POI to be approximately 3.7% (Golezar et al., 2019). POI has a multifactorial etiology involving genetic susceptibility, medical gonadotoxic injury, autoimmunity, and infections (Chen et al., 2026). Growing evidence suggests that chronic inflammation contributes to the development and progression of POI by accelerating ovarian fibrosis, follicle depletion, and functional decline (Chen et al., 2026). Beyond infertility, POI/premature ovarian failure poses substantial risks to women’s systemic health because chronic hypoestrogenism is associated with vasomotor and urogenital symptoms, reduced bone mineral density or osteoporosis, increased cardiovascular risk, neurocognitive and mood disturbances, sexual dysfunction, and higher long-term morbidity or mortality (Faubion et al., 2015).

The pathogenesis of POI remains incompletely understood, but it may be associated with defects in the establishment of the primordial follicle pool, abnormal follicle recruitment or maturation, and accelerated follicular atresia. Follicular atresia is mainly driven by granulosa cell death pathways, including autophagy, necrosis, apoptosis, and ferroptosis (Liu et al., 2023). Granulosa cell dysfunction is closely linked to both diminished ovarian reserve and POI (Liu and Fang, 2025). In these disorders, granulosa cells show downregulation of key glycolytic enzymes such as HK2, PKM2, and LDHA, leading to reduced glycolytic activity and decreased lactate production. This metabolic impairment is accompanied by increased apoptosis, which weakens the capacity of granulosa cells to support follicular development (Li et al., 2023; Li W. et al., 2024; Zhu Q. et al., 2025).

AARS2-mediated lactylation has been identified as a pathogenic metabolic mechanism in POI. Serum lactate and free fatty acids are elevated in POI patients and are negatively associated with ovarian reserve. In granulosa cells, AARS2 overexpression or AARS2 R199C mutation enhances lactyltransferase activity and increases lactylation of pyruvate dehydrogenase E1 subunit alpha 1 (PDHA1) and carnitine palmitoyltransferase 2 (CPT2). Mechanistically, PDHA1 lactylation suppresses oxidative metabolism and activates mTORC1-dependent granulosa-cell proliferation, whereas CPT2 lactylation impairs fatty acid oxidation and potentiates FSH signaling through the PPARγ pathway (Zhang Z. L. et al., 2025). An Aars2 R194C knock-in mouse model, which mimics human AARS2 R199C, further confirmed that this single mutation is sufficient to induce POI-like phenotypes *in vivo* (Zhang H. H. et al., 2026). Conversely, β-alanine-mediated inhibition of AARS2-catalyzed lactylation attenuated these POI-like phenotypes (Zhang Z. L. et al., 2025). Collectively, these findings suggest that a balanced glycolysis–lactate–lactylation axis in granulosa cells is essential for maintaining follicle homeostasis. Lactate/lactylation inhibition may provide a potential strategy for POI prevention or treatment.

### 3.5 Lactylation and reproductive system tumors

Malignant tumors typically exhibit the Warburg effect, resulting in the massive accumulation of lactate. Lactate-derived lysine lactylation has been identified as a critical metabolic-epigenetic bridge that links glycolytic flux to malignant progression through modifications of both histones and non-histone proteins, such as transcription factors, metabolic enzymes, and DNA repair enzymes (Mi et al., 2025; Sun et al., 2025). Lactylation drives hallmark cancer phenotypes, including sustained proliferation, invasion and metastasis, stemness maintenance, immune evasion, therapy resistance, and metabolic plasticity (Ji and Xia, 2025). These effects are mediated by various downstream mechanisms, including epigenetic activation of oncogenic programs, dysregulation of key signaling pathways, EMT, and tumor microenvironment remodeling (Huang C. et al., 2024). Importantly, lactylation often establishes self-reinforcing feedback loops that reinforce the malignant state (Wei S. et al., 2024). Below, we systematically summarize the specific sites of lactylation and regulatory mechanisms in reproductive system cancers, underscoring their potential as therapeutic targets and prognostic biomarkers.

#### 3.5.1 Ovarian cancer

Ovarian cancer is the eighth most prevalent female tumor and the main cause of mortality among gynecological cancers, with over 310,000 new cases reported worldwide in 2022 (Bray et al., 2024). Despite advances in surgical and chemotherapeutic strategies, patient outcomes remain poor due to late-stage diagnosis and resistance to chemotherapy, with a 5-year relative survival rate less than 50% (Fu et al., 2024). Clinical and multi-omics evidence suggests that lysine lactylation is a key metabolic-epigenetic mechanism that drives ovarian cancer progression, immune evasion, and therapeutic failure. Clinically, lactylation levels are correlated to advanced tumor stage, elevated CA125 levels, ascites, and poor survival (Zhou et al., 2026). Histone lactylation modifications such as H3K18la and H3K9la are associated with aggressive pathology and reduced survival, and may serve as independent prognostic factors (Sun et al., 2025; Chao et al., 2024). Single-cell transcriptomics analyses further reveal that chemoresistant cell subsets exhibit elevated glycolysis and higher global lactylation levels (Ren F. et al., 2025). Lactylation-related gene signatures not only effectively stratify ovarian cancer patients into distinct molecular and prognostic subtypes, but also delineate the tumor immune landscape, predict responsiveness to both immunotherapy and chemotherapy (Luan et al., 2026).

Lactate-driven lactylation establishes a positive feedback loop during metabolic reprogramming, thereby amplifying lactate synthesis and promoting ovarian cancer progression. For instance, hypoxia-induced lactylation of phosphofructokinase platelet type (PFKP) stabilizes this glycolytic enzyme, enhances glycolytic flux, and promotes tumor growth by suppressing PTEN (Mi et al., 2025). Ovarian cancer cells also intrinsically amplify glycolysis through the overexpression of cancer-testis antigen LDHC4. This leads to increased H4K12 lactylation at the phosphoglycerate kinase 1 (PGK1) promoter and upregulates its expression, which drives glycolytic flux and promotes ovarian cancer progression (Lin et al., 2026). This glycolysis–lactylation axis may be therapeutically exploitable, as Tanshinone I suppresses ovarian cancer growth by inhibiting Forkhead box protein K1/K2 (FOXK1/FOXK2)-dependent glycolysis, reducing lactate production, and selectively decreasing H3K18la (Jin et al., 2025).

Lactylation also contributes to treatment resistance by enhancing DNA repair. After platinum exposure, glucose deprivation promotes ACAT1-mediated acetylation of malate enzyme 2 (ME2) at K156, which redirects glutamine-derived malate toward lactate production and supports lactylation of homologous recombination repair proteins meiotic recombination 11 (MRE11) and nijmegen breakage syndrome 1 (NBS1) thereby facilitating DNA repair and drug resistance (Zheng et al., 2025). In platinum-resistant ovarian cancer, GCN5 promotes both H3K9 lactylation and repair protein RAD51 K73 lactylation. This dual lactylation upregulates the transcription of RAD51 and homologous recombination repair genes, resulting in DNA damage repair and reducing cisplatin sensitivity (Sun et al., 2025). In PARP inhibitor resistance, lactate-induced H4K12la activates MYC-dependent super-enhancer regulation of RAD23A, which enhances nucleotide excision repair and confers niraparib resistance (Lu B. et al., 2025).

Ferroptosis is closely involved in ovarian cancer progression and treatment response, as dysregulated iron metabolism, intracellular iron overload, and lipid ROS accumulation can shape tumor proliferation, metastasis, and sensitivity to therapy (Wang et al., 2021). Lactylation further modulates ovarian cancer cell survival by suppressing ferroptotic cell death. Mechanistically, H3K18la is enriched at the TRA2A promoter and upregulates TRA2A expression. TRA2A then promotes alternative splicing of STIL toward the STIL-L isoform, which alters iron metabolism, inhibiting ferroptosis, and ultimately reducing cisplatin sensitivity (Gao M. et al., 2025).

Lactylation also reprograms the tumor microenvironment to promote immunosuppression and metastasis in ovarian cancer. Tumor-derived lactate acts via the macrophage receptor GPR132 to induce H3K18la at the CCL18 promoter, driving M2 macrophage polarization and tumor progression (Sun J. et al., 2024). Furthermore, LDHB-driven lactate production elevates H3K18 lactylation at the PD-L1 promoter, leading to T-cell dysfunction and immune escape (Hu et al., 2025b). Transcription factor FOXK1-driven glycolysis contributes to immune evasion in high-grade serous ovarian cancer by promoting lactylation of the thymocyte selection-associated high mobility group box (TOX) protein and subsequent CD8^+^ T cell exhaustion (Li, 2026).

These findings underscore that lactylation connects metabolic rewiring, DNA repair, chemoresistance, ferroptosis suppression, immune escape, and therapeutic resistance into an integrated pathogenic network in ovarian cancer. Targeting lactylation-related pathways may offer a promising strategy to inhibit tumor proliferation, overcome therapeutic resistance, and promote ovarian cancer cell death.

#### 3.5.2 Cervical cancer

**FIGURE 4:**
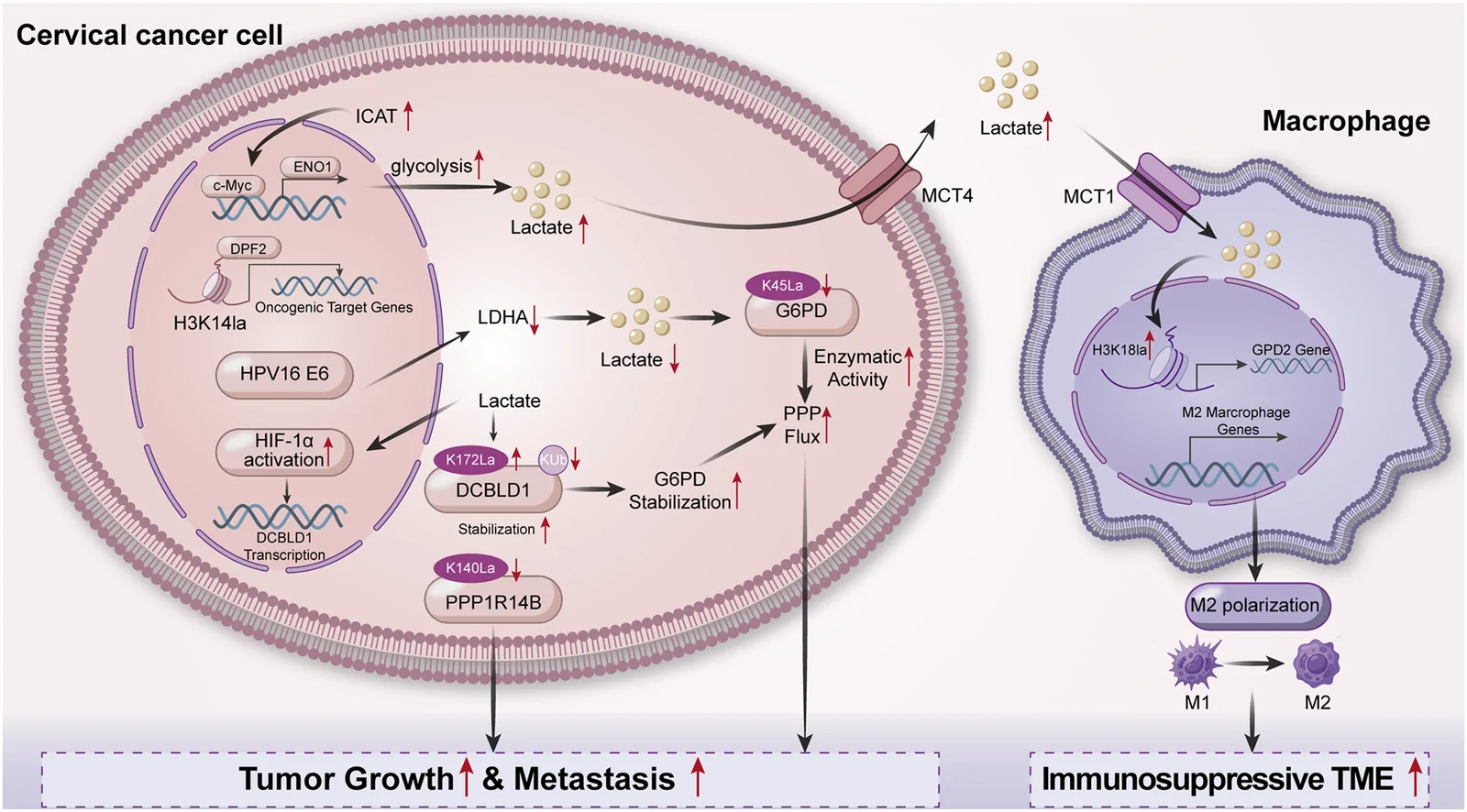
Lactylation in cervical cancer progression and TME remodeling. Aberrant lactate metabolism drives epigenetic and post-translational modifications in both tumor cells and the TME. Dysregulated lactylation is associated with metabolic reprogramming, oncogenic transcription, immune cell polarization, and immunosuppressive microenvironment formation, thereby contributing to tumor growth, invasion, and metastasis.

Cervical cancer is the most prevalent gynecological cancer, with more than 660,000 new cases and 350,000 deaths annually (Bray et al., 2024). Its development is primarily caused by persistent infection with high-risk human papillomavirus (HPV), particularly HPV16 and HPV18 (Schiffman et al., 2016). Recurrence and therapy resistance are major clinical challenges in the treatment of cervical cancer (Ferrall et al., 2021). A quantitative lactylome study in HeLa cells identified 3,633 lactylation sites on more than 1,600 proteins, and stable lactylated substrates were enriched in genetic information processing, metabolism, RNA metabolism, cell cycle, and DNA repair (He et al., 2024). These findings suggest that lactylation may represent a regulatory mechanism that links metabolic reprogramming to cervical cancer growth (Figure 4).

Non-histone lactylation participates in metabolic reprogramming in cervical cancer, especially through the glucose-6-phosphate dehydrogenase (G6PD), the core metabolic enzyme of the pentose phosphate pathway (PPP). Lactate increases the expression of Discoidin, CUB, and LCCL domain-containing type 1 (DCBLD1) by promoting HIF-1α enrichment at the DCBLD1 promoter. It also stabilizes DCBLD1 through K172 lactylation. Increased DCBLD1 prevents autophagic degradation of G6PD, thereby sustaining G6PD expression and oxidative PPP activity. This increases the levels of reduced nicotinamide adenine dinucleotide phosphate (NADPH) and GSH, decreases ROS, and promotes cervical cancer cell proliferation, migration, invasion, and xenograft growth (Meng et al., 2024a). Another PPP-related mechanism involves HPV16, a major etiological factor in cervical cancer. HPV16 E6 directly intersects with this metabolic control by suppressing LDHA and reducing inhibitory lactylation of G6PD at K45, thereby increasing G6PD activity and supporting proliferation (Meng et al., 2024b).

Lactylation-related genes also have prognostic and immunological relevance. Based on these genes, cervical cancers were classified into two molecular clusters, and a two-gene prognostic model containing ISY1 and PPP1R14B was established. PPP1R14B expression was negatively correlated with CD8^+^ T-cell infiltration. In addition, mutation of PPP1R14B K140 reduced its lactylation and enhanced cervical cancer cell proliferation and migration, suggesting that non-histone lactylation may influence both tumor behavior and immune contexture (He et al., 2024).

Identifying specific lactylation-modifying enzymes is crucial for the development of targeted therapies. Recent mechanistic work has identified DPF2 as a reader of H3K14la in cervical cancer. DPF2 binds H3K14la through its DPF domain and co-localizes with H3K14la at promoters of tumor-related genes such as SEMA5A, ROCK1, and SOAT1. Disruption of the DPF2–H3K14la interaction reduces oncogenic gene transcription, cell proliferation, and colony formation (Zhai et al., 2024).

Lactylation further remodels the cervical cancer immune microenvironment by regulating tumor-associated macrophages (TAMs). During the transition from normal cervical epithelium to low-grade squamous intraepithelial lesions, high-grade squamous intraepithelial lesions, and cervical squamous cell carcinoma, H3K18la levels and M2 macrophage infiltration progressively increase. Cervical cancer cell-derived lactate enters macrophages through MCT1 and elevates H3K18la. This process suppresses M1 markers such as CD86 and iNOS, while increasing M2 markers such as CD206 and ARG1. Glycerol-3-phosphate dehydrogenase 2 (GPD2) functions as a histone lactylation-regulated target, and GPD2 knockdown reversed lactate-induced M2 polarization (Huang C. et al., 2024). A related tumor–macrophage lactylation pathway is mediated by the inhibitor of β-catenin and T-cell factor (ICAT). ICAT is upregulated in cervical cancer and promotes c-Myc nuclear translocation, which enhances ENO1 transcription and increases glycolysis-dependent lactate production. Tumor-derived lactate then induces pan-histone lactylation and H3K18la in TAMs, activates H3K18la enrichment at the ARG1 promoter, and drives M2-like polarization (Dang et al., 2025).

Together, these studies indicate that lactylation contributes to cervical cancer through both tumor-cell-intrinsic and microenvironmental mechanisms. Larger patient cohorts and deeper mechanistic studies are still needed before these findings can be advanced toward clinical translation.

#### 3.5.3 Endometrial cancer

The incidence of endometrial cancer (EC) is increasing annually. A hallmark of EC is metabolic reprogramming toward aerobic glycolysis, which causes substantial lactate accumulation and supports tumor aggressiveness (Giatromanolaki et al., 2006; Han et al., 2015; Shi et al., 2023). Clinically, high lactylation-related risk scores are correlated with poorer survival, distinct clinicopathological features, reduced immune infiltration, and differential responses to therapy, highlighting their prognostic and predictive relevance (Chen L. et al., 2025; Gu et al., 2025; Yin and Luo, 2025). These findings suggest that lactylation is not only a metabolic consequence but also a disease-relevant regulatory mechanism in EC.

Lactylation creates a self-reinforcing circuit between cellular metabolism and epigenetic regulation. H3K18la transcriptionally activates ubiquitin-specific peptidase 39 (USP39), which acts as an oncogenic factor in EC. USP39 then stabilizes glycolytic enzyme PGK1 through deubiquitination, thereby activating the PI3K–AKT–HIF-1α pathway. This cascade enhances glycolysis-related proteins, including GLUT1, HK2, LDHA, MCT1, and MCT4. This forms a positive feedback loop in which glycolysis-derived lactate enhances histone lactylation, while lactylation further promotes glycolysis and malignant progression (Wei S. et al., 2024). Concurrently, lactate directly lactylates the rate-limiting glycolytic enzyme phosphofructokinase muscle type (PFKM) at K678. This modification enhances PFKM activity, lactate production, EC cell proliferation, invasion, and angiogenesis (Wang B. et al., 2025).

Hypoxia further links lactylation to EC metabolic plasticity and aggressive EC phenotypes. Under hypoxic conditions, EC cells show increased glycolysis and lactate accumulation. Lactate elevates histone H3K18 lactylation enrichment at the promoter of the solute carrier family 9 member A7/sodium-hydrogen exchanger 7 (SLC9A7/NHE7), which directly activates NHE7 transcription. NHE7 subsequently upregulates cytochrome c oxidase subunit 6C, enhances oxidative phosphorylation, and triggers endoplasmic reticulum stress, thereby promoting EC progression (Yang et al., 2026a).

Lactate-mediated lactylation also remodels the EC tumor microenvironment by regulating macrophage function. Tumor-derived lactate is imported into macrophages through MCT1 and induces H3K18la enrichment at the DNA methyltransferase 1 (DNMT1) promoter, thereby increasing DNMT1 expression. DNMT1 then methylates and silences NHE7 in macrophages, which activates MAPK signaling and promotes macrophage senescence and M2 polarization. These dysfunctional macrophages create a tumor-promoting microenvironment and enhance EC cell proliferation, migration, invasion, and xenograft tumor growth (Yang et al., 2026b).

In endometrial cancer, both histone and non-histone lactylation contribute to disease progression by coordinating glycolytic reprogramming, hypoxia adaptation, endoplasmic reticulum stress, and immune suppression. Therapeutic strategies that target lactylation-related signaling or lactate-mediated tumor–macrophage communication may offer new opportunities for EC treatment.

## 4 Therapies targeting lactate metabolism and lactylation

Targeting lactate metabolism and lactylation has emerged as a promising therapeutic direction, particularly in settings characterized by excessive lactate accumulation or aberrant lactylation-dependent transcriptional reprogramming. In principle, therapeutic intervention can be achieved by reducing lactate production, blocking lactate transport, or directly modulating the enzymatic machinery and target sites of lactylation. Several clinically approved drugs possess the therapeutic potential to modulate lactate production and lactylation dynamics. For instance, metformin modulates lactate production via mitochondrial complex I inhibition and acts as an epigenetic modulator by enhancing antiviral immunity through IRF9 lysine lactylation (Abdel-Wahab et al., 2019; Miao et al., 2026). Here, we summarize these approaches and their applications in reproductive system diseases (Table 3).

**TABLE 3:** Representative examples of therapy strategies targeting lactate metabolism and lactylation in the reproductive system.

Target category	Target	Drug	Disease	Effect	Ref
Inhibit lactate production	HK	2-DG	Cervical cancer/Endometrial carcinoma/Ovarian cancer/Endometriosis	Inhibit cell proliferation, migration, and invasion, induce apoptosis	Wen et al. (2024), Sun et al. (2025), Wei S. et al. (2024), Lu B. et al. (2025), Dang et al. (2025)
			Preeclampsia	Attenuate hypoxia-induced pyroptosis	Xu J. et al. (2025)
Promote lactate production	PKM2	TEPP-46	Preeclampsia; PCOS	Promote trophoblast proliferation; Inhibit nuclear PKM2 and PCOS-like phenotype	Xu R. et al. (2025), Yu et al. (2025a)
Inhibit lactate production	LDH	Oxamate	Endometrial carcinoma/Cervical cancer	Inhibit proliferation, migration; Reverse M2 polarization	Huang C. et al. (2024), Wei S. et al. (2024), Yang et al. (2026b)
			Preeclampsia	Attenuate fibrosis	Li et al. (2022)
			PCOS	Increase uterine receptivity	Shan et al. (2026)
Inhibit lactate production	LDH	FX-11	Ovarian cancer	Enhance the sensitivity to Cisplatin	Sun et al. (2025)
Inhibit lactate production	LDH	AXKO-0046	Ovarian cancer	Enhance the killing effect of T cells	Hu et al. (2025b)
Inhibit lactate transport	MCT	CHC	POI	Inhibit AARS2-overexpressing induced GC proliferation	Zhang Z. L. et al. (2025)
Inhibit lactate transport	MCT1	AZD3965	Pregnancy loss	Rescue pregnancy in abortion-prone mouse model	Gao et al. (2022)
Inhibit lactate transport	MCT1	SR13800	Cervical cancer	Inhibit M2 polarization	Huang C. et al. (2024)
Regulate writers	GCN5	MB-3	Ovarian cancer	Enhance the sensitivity to chemotherapy	Sun et al. (2025)
Regulate writers	AARS2	β-alanine	POI	Increase primordial follicle reserve	Zhang Z. L. et al. (2025)
Inhibit lactylation	H4K12la	Elbasvir	Ovarian cancer	Suppress tumor growth, downregulate H4K12la and Ki-67 expression	Lin et al. (2026)
Exogenous lactate	Lactate	Lactate supplementation	Oocyte maturation and early embryogenesis; Endometrial receptivity	Increase oocyte maturation rate and enhance early embryo development; Physiological concentration enhances embryo attachment; high concentration impairs attachment	Yang Q. et al. (2022), Yang D. et al. (2024)

### 4.1 Modulating lactate production

Since lactate is the direct substrate for lactylation, reducing its production is the most direct strategy for suppressing lactylation. Currently, small-molecule compounds targeting key enzymes involved in lactate biosynthesis have been employed to inhibit intracellular lactate production and advance related studies on lactylation, with potential as therapeutic agents targeting lactylation modifications. In research on reproductive system-related disorders, for example, in preclinical models of cervical cancer, ovarian cancer, and preeclampsia, HK inhibitor 2-DG suppresses glycolysis, reduces lactate levels, and normalizes key cellular processes such as proliferation and migration (Liang et al., 2025; Dang et al., 2025). The lactylation of the lysine residue at position 169 of lactate-induced synaptosomal-associated protein 29 (SNAP29) promotes its degradation, impairing macroautophagy/autophagy and trophoblast cell function, ultimately leading to early pregnancy loss. In contrast, 2-DG reduces lactate levels while reversing this process, suggesting potential therapeutic benefits for recurrent miscarriage (Lu et al., 2026). Pharmacological activation of PKM2 with the agonist TEPP-46 promotes glycolysis and restores the proliferative capacity of trophoblast cells in preeclampsia (Xu R. et al., 2025). In PCOS, TEPP-46 promotes the formation of PKM2 tetramers, preventing its entry into the cell nucleus and thereby inhibiting pathological histone lactylation (Yu et al., 2025a).

Directly targeting LDH is another promising strategy to limit lactate production. Stiripentol, a clinically approved anti-epileptic drug with LDHA inhibitory activity, can also suppress lactylation (Chen H. et al., 2024). In ovarian cancer, FX-11 enhances sensitivity to cisplatin (Sun et al., 2025). AXKO-0046 exerts anti-tumor immune effects by enhancing T-cell-mediated killing (Hu et al., 2025b). Oxamate primarily exerts its effect by inhibiting lactate dehydrogenase, the enzyme that converts pyruvate to lactate during glycolysis, thereby blocking the Warburg effect and reducing protein lactylation levels (Stepanov et al., 2026). The administration of oxamate intervention reduced levels of endometrial lactate and H3K18la, decreased ERα expression, and successfully reversed implantation failure in PCOS model mice (Shan et al., 2026).

Interventions that alter pyruvate utilization can also influence lactylation. By promoting the conversion of pyruvate to acetyl-CoA, pyruvate dehydrogenase kinase inhibition reduces lactate generation and suppresses downstream lactylation. Sodium dichloroacetate (DCA) is a representative example of this strategy and has shown efficacy in reducing H3K18 lactylation in prostate cancer models (He et al., 2023).

### 4.2 Blocking lactate transport

Since lactate transport also influences intracellular lactate levels and the degree of lactylation modification, small molecules targeting the lactate transport system hold promise as potential drugs for regulating lactylation modification. Accordingly, blockade of key molecules involved in lactate transport is a viable approach. Therefore, MCTs represent a second major target for modulating lactylation. However, research on MCT inhibitors in the treatment of reproductive system-related diseases remains highly limited. Current experimental studies have shown that MCT inhibitors can attenuate pathological lactylation and have also shown disease-specific benefit. For example, recent studies have demonstrated that lactate metabolism plays a critical role in RPL mediated by decidual macrophages via the HIF-1α/SRC/LDHA pathway, and administration of AZD3965 (an MCT1 inhibitor) can reverse pregnancy loss in RPL model mice—an effect likely associated with inhibition of lactylation and promotion of an anti-inflammatory phenotypic polarization in placental macrophages (Gao et al., 2022). Another MCT1 inhibitor, α-cyano-4-hydroxycinnamic acid (CHC), has been shown to reduce mitochondrial lactate levels in mouse ovaries, thereby delaying excessive activation and depletion of follicles, demonstrating potential therapeutic effects in an early-onset ovarian failure model (Huang C. et al., 2024; Zhang Z. L. et al., 2025; Gao et al., 2022).

### 4.3 Targeting the lactylation enzymes and specific sites

A more direct strategy is to interfere with the molecular machinery responsible for generating, installing, removing, or recognizing lactylation. Using a target-blocking peptide or siRNA to disrupt the ACSS2- or GTPSCS-related pathways can reduce intracellular lactyl-CoA availability and thereby suppress lactylation (Zhu R. et al., 2025; Wu G. et al., 2025).

Direct pharmacological targeting of “writers” enzymes offers a precise strategy to modulate pathogenic lactylation. For KATs, the p300 inhibitors C646 (Wu et al., 2024) and A485 (Yang et al., 2023), as well as the TIP60 inhibitor MG149 (Jia et al., 2023) effectively suppress lactylation. The GCN5 inhibitor MB-3 enhances sensitivity to platinum chemotherapy in ovarian cancer (Sun et al., 2025). Lactate analog β-alanine competitively interferes with lactate binding to AARS1 and inhibits lactylation of oncogenic proteins (Zong et al., 2024). β-alanine also effectively inhibits the AARS2 and restores ovarian follicular reserve (Zhang Z. L. et al., 2025).

Therapies targeting lactylation “erasers” HDACs and SIRTs that catalyze the removal of lactyl groups from lysine residues are another strategy for targeting lactylation. The HDAC2 inhibitor tucidinostat preserves METTL3 lactylation and enhances cisplatin therapy sensitivity in triple-negative breast cancer (He et al., 2025). The SIRT3 activator honokiol decreases lactylation levels and induces hepatocellular carcinoma cell apoptosis (Jin et al., 2023).

With respect to lactylation sites, Elbasvir, a compound originally developed as a hepatitis C virus nonstructural protein 5A inhibitor, has been identified as an inhibitor of H4K12 lactylation and suppresses ovarian tumor growth *in vivo* (Lin et al., 2026). Similarly, the FDA-approved glucocorticoid dexamethasone was found to act as a pharmacological suppressor of H4K12la, and this property boosts the antitumor efficacy of the HIF-2α inhibitor belzutifan in clear cell renal cell carcinoma lacking von Hippel‒Lindau (VHL) function (Zhan et al., 2026).

### 4.4 Physiological and metabolic modulation strategies

Beyond classical pharmacological inhibition, lactylation can also be modulated through physiological and metabolic strategies. Exercise represents a lifestyle-based metabolic intervention that alters systemic and tissue lactate flux, thereby affecting lactylation-dependent cellular responses. In high-fat diet-induced obese rats, exercise, particularly high-intensity interval training, restored testicular lactate and LDH levels, suggesting that exercise can improve reproductive metabolic coupling (Maleki et al., 2024).

ART offers a unique *ex vivo* platform in which gametes, embryos, and reproductive tissue models can be cultured under defined conditions. This allows lactate availability, glucose–pyruvate balance, oxygen tension, pH, and lactate transport to be adjusted to fine-tune stage-specific lactylation without systemic exposure. Thus, exogenous lactate supplementation is better viewed as culture-medium optimization. It may benefit oocyte maturation and early embryo development, but its effects are concentration- and stage-dependent and may become harmful when excessive (Yang Q. et al., 2022; Yang D. et al., 2024).

In studies on recurrent miscarriage, the metabolic modulator metformin or the anti-aging combination dasatinib + quercetin significantly improved the placental metabolic microenvironment by influencing lactate-mediated metabolic reprogramming, reducing SNAP29 lactylation levels, and restoring autophagy flux, thereby effectively reversing placental developmental defects and decreasing the incidence of miscarriage in mice (Lu et al., 2026). However, current research indicates that metformin is a relatively definitive lactate-producing agent. Its fundamental mechanism involves inhibiting mitochondrial respiratory chain complex I, thereby impairing oxidative phosphorylation and elevating the NADH/NAD^+^ ratio; consequently, pyruvate cannot enter the TCA cycle and is reduced to lactate by LDH. Additionally, metformin suppresses hepatic gluconeogenesis and reduces lactate clearance, collectively leading to lactate accumulation (DeFronzo et al., 2016; Maurer et al., 2023; Foretz et al., 2010). Therefore, metformin is more likely to promote the Warburg effect, which appears inconsistent with some current research findings suggesting that metformin exerts its antitumor effects by inhibiting lactylation modifications. As a potential regulator of lactylation modifications, metformin’s specific impact and mechanisms regarding protein lactylation require further investigation for validation.

### 4.5 Clinical translation of lactylation-pathway modulators

Although most of the known small-molecule drugs capable of regulating lactate metabolism and lactylation are currently only utilized in basic experimental studies, several agents have already advanced into clinical evaluation (Table 4). Among compounds targeting lactate production, the PDK inhibitor DCA has completed phase II evaluation in brain cancer (Clinical trial: NCT00540176), while the HK inhibitor 2-DG has been evaluated in a phase I trial in advanced solid tumors (Clinical trial: NCT00096707). In addition, stiripentol, a clinically approved antiseizure drug with LDHA-inhibitory activity, has entered a registered phase II study for peritoneal metastatic carcinoma (Clinical trial: ChiCTR2400083649). In parallel, the MCT1 inhibitor AZD3965 has completed first-in-human phase I evaluation in advanced cancer and lymphoma (Clinical trial: NCT01791595). Clinical translation has likewise extended to regulators of lactylation itself. Several writer- and eraser-directed agents, particularly those acting on CBP/p300 or HDAC family proteins, have entered clinical studies. Although preclinical studies indicate that some of these compounds can influence lactylation, no interventional trial to date has prospectively incorporated lactylation as a predefined pharmacodynamic or biomarker endpoint. Future trials should incorporate tissue Kla measurements, site-specific lactylation readouts, or lactylation-responsive gene signatures to establish whether clinical activity is truly mediated through lactylation remodeling. However, current clinical translation studies on lactylation-modified targeted drugs are predominantly focused on the treatment of oncological diseases, while research in other conditions—particularly those related to the reproductive system, such as RPL, PCOS, and preeclampsia—remains limited. As lactylation modification continues to be explored more extensively in reproductive system disorders, we anticipate that these lactylation-modified targeted drugs will demonstrate significant therapeutic potential in treating such conditions.

**TABLE 4:** Clinical trials targeting the modulation of lactate metabolism and lactylation.

Effects	Therapeutic targets	Drugs	Clinical phase	Diseases	Trial No.
Lactate production	PDK	DCA	Phase II	Brain Cancer	NCT00540176
Lactate production	HK	2-DG	Phase I	Advanced Solid Tumors	NCT00096707
Lactate production	LDHA	Stiripentol	Phase II	Peritoneal Metastatic Carcinoma	ChiCTR2400083649
Lactate uptake	MCT1	AZD3965	Phase I	Diffuse Large B-cell Lymphoma and Burkitt’s Lymphoma	NCT01791595
Writer	CBP/p300	Curcumin	Phase II	Breast Cancer	NCT01740323
Writer	CBP/p300	FT-7051	Phase I	Metastatic Castration-Resistant Prostate Cancer	NCT04575766
Writer	CBP/p300	CCS1477 (Inobrodib)	Phase I/II	Advanced Solid Tumors	NCT03568656
Writer	Dual BET + CBP/p300	EP31670 (NEO2734)	Phase I	Advanced Solid Tumors and Hematological Malignancies	NCT05488548
Eraser	HDAC	VTR-297 (Trichostatin A)	Phase I	Hematologic Malignancies	NCT03838926
Eraser	HDAC	MS-275 (Entinostat)	Phase II	Cholangiocarcinoma and Pancreatic Adenocarcinoma	NCT03250273
Eraser	HDAC	Tucidinostat	Phase III/Approved	Diffuse large B-cell lymphoma	NCT04231448
Eraser	HDAC	Honokiol	Phase I	Non-Small Cell Lung Cancer	NCT06566443
Exogenous lactate	Lactate	0.5 M Na Lactate	Phase II	Acute Heart Failure	NCT01981655

## 5 Challenges and future perspectives

**FIGURE 5:**
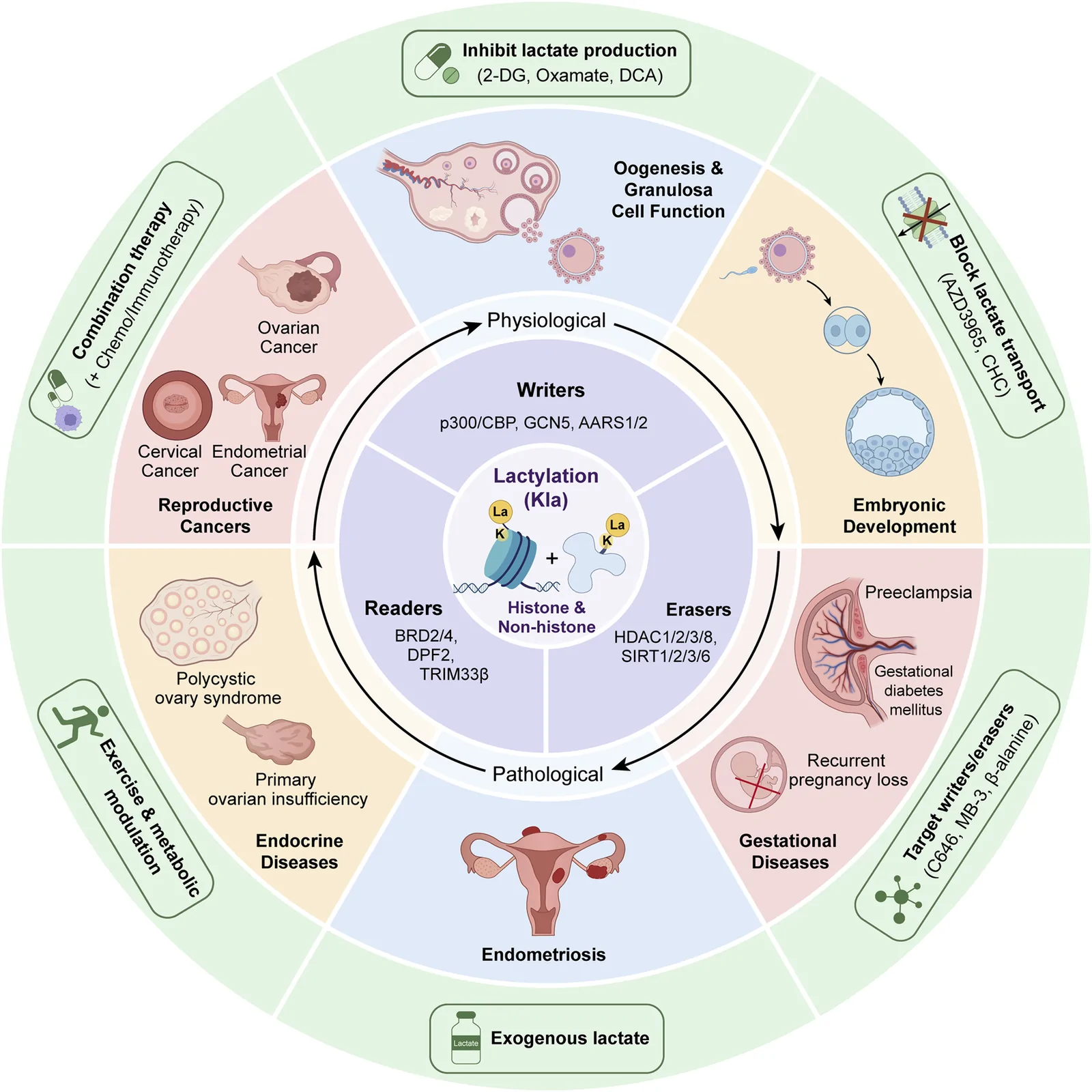
Lactylation-centered regulatory network and therapeutic landscape in reproductive health and disease. The metabolic-epigenetic axis mediated by protein lactylation (Kla) is dynamically governed by a specific network of writers, readers, and erasers. This modification coordinates normal reproductive physiological functions while simultaneously driving the pathogenesis of reproductive disorders. Consequently, targeting this regulatory network provides a promising therapeutic strategy for reproductive pathologies.

Protein lactylation is a lysine PTM first reported in 2019 (Zhang et al., 2019). It is now regarded as an important mechanism linking cellular metabolism to epigenetic regulation and protein function. Lactylation participates in diverse biological processes, including metabolic adaptation, transcription, cell death, immune responses, and tissue remodeling. Clinical findings and experimental data have linked lactylation in a wide range of human diseases, such as cancer, cardiovascular disease, musculoskeletal disorders, and neurological disorders. This review systematically summarizes the roles of lactylation in the reproductive system, covering physiological processes such as gametogenesis, early embryonic development, decidualization, implantation, and placentation, as well as pathological diseases, including gestational diseases, reproductive endocrine disorders, benign gynecological diseases, and reproductive tumors. These findings suggest that lactylation may serve as a biomarker and therapeutic target in reproductive medicine (Figure 5).

Although lactylation research in the reproductive system has advanced, it remains at an early stage. A major challenge is its strong context dependency. Lactylation patterns vary across reproductive organs, cell types, and developmental stages, whereas most studies still provide static observations under a single condition. Cellular heterogeneity further complicates interpretation, as reproductive tissues and the tumor microenvironment contain diverse populations, including oocytes, granulosa cells, trophoblast subtypes, decidual cells, stromal cells, endothelial cells, immune cells, and cancer cells. Future studies should therefore characterize lactylation dynamics across the menstrual cycle, implantation window, and gestational stages. Single-cell transcriptomic and epigenomic approaches may help define cell-specific lactylation patterns and guide the design of combination immunotherapy strategies tailored to distinct cellular contexts.

Methodological limitations also restrict progress in this field. The specificity of currently available anti-Kla antibodies remains suboptimal because of potential cross-reactivity with structurally related lysine modifications. Therefore, more sensitive and more specific detection tools are needed, including site-specific antibodies, fluorescent probes, high-resolution LC–MS/MS, and electrochemical assays. In parallel, the interplay between lactylation and other PTMs also requires further study, especially because Kla and Kac may share lysine residues and regulatory enzymes (Yu et al., 2025b). Furthermore, the identification of specific lactylation “writers”, “erasers”, and “readers” will be essential for the development of selective therapeutic modulators (Wang X. et al., 2025), although their precise roles in the reproductive system are still incompletely understood. Future progress will depend on integrative approaches, including higher-resolution proteomics, top-down mass spectrometry techniques to identify co-occurring modifications (Poncha et al., 2025), and site-specific mutagenesis tools such as genetic code expansion (Osgood et al., 2025). Organoid systems that mimic physiological microenvironments may also be used for high-throughput screening of modulating compounds.

Clinical translation remains challenging because lactylation acts differently across biological settings. In physiological conditions, lactylation supports normal reproductive functions, whereas dysregulated lactylation may drive disease. Therefore, therapeutic strategies should target disease-specific lactylation sites, pathogenic substrates, or cell populations rather than globally inhibiting Kla. Current evidence for lactylation-targeted therapy is still mainly derived from cancer research. In reproductive tumors, the associations between lactylation and clinicopathological features require validation in larger independent cohorts. For non-malignant reproductive diseases, lactylation-based treatment may need to be combined with endocrine, anti-inflammatory, antifibrotic, or immune-modulatory therapies. Selective inhibitors remain limited, and off-target effects are a major concern, highlighting the need for precision delivery. Taking advantage of the anatomical accessibility of the reproductive tract, local approaches such as ultrasound-guided intraovarian injection and minimally invasive intrauterine infusion may provide convenient and site-directed routes for precise modulation of lactate-lactylation signaling (Zhao et al., 2026). In addition, tissue-targeted platforms, including nanoparticles, exosomes, and hydrogels, may enable localized control of lactate availability or lactylation activity while minimizing systemic toxicity (Abbasi et al., 2026). Safety assessments of lactylation-targeted drugs are required to evaluate fertility preservation, pregnancy-related risk, and long-term endocrine effects.

## 6 Conclusion

Lactylation represents a frontier in reproductive medicine that links metabolic state, epigenetic regulation, and cellular function in a clear biochemical pathway. This modification provides a new framework for understanding fertility, reproductive development, and the pathogenesis of reproductive disorders. A deeper mechanistic understanding of lactylation may improve diagnosis, risk stratification, and targeted interventions of female reproductive disorders, while preserving the physiological functions.

## Glossary

2-DG: 2-Deoxy-D-glucose
AARS1/2: Alanyl-tRNA synthetase 1/2
ACSS2: Acetyl-CoA synthetase 2
ART: Assisted reproductive technology
ATP: Adenosine triphosphate
BCAT2: Branched chain amino acid transaminase 2
BRD: Bromodomain-containing protein
cAMP: cyclic adenosine monophosphate
CBP: CREB-binding protein
CHC: α-cyano-4-hydroxycinnamic acid
CPT2: Carnitine palmitoyltransferase 2
CREB: cAMP response element-binding protein
CTPS1: CTP synthase 1
DCA: Dichloroacetate
DCBLD1: Discoidin, CUB, and LCCL domain-containing type 1
DNMT1: DNA methyltransferase 1
Drp1: Dynamin-related protein 1
EC: Endometrial cancer
EMS: Endometriosis
EMT: Epithelial-mesenchymal transition
ENO1: Enolase 1
FOXK1/K2: Forkhead box protein K1/K2
FOXM1: Forkhead box protein M1
FSH: Follicle-stimulating hormone
G6PD: Glucose-6-phosphate dehydrogenase
GADD45A: Growth arrest and DNA damage inducible alpha
GCs: Granulosa cells
GDM: Gestational diabetes mellitus
GLO1/2: Glyoxalase 1/2
GPD2: Glycerol-3-phosphate dehydrogenase 2
GPR: G protein-coupled receptor
GPX4: Glutathione peroxidase 4
GSH: Glutathione
GTP: Guanosine triphosphate
GTPSCS: Guanosine triphosphate-specific succinyl-CoA synthetase
GV: Germinal vesicle
GVBD: Germinal vesicle breakdown
hCG: Human chorionic gonadotropin
HDACs: Histone deacetylases
HIF: Hypoxia-inducible factor
HK: Hexokinase
HLA-F: Human leukocyte antigen F
HMGB1: High mobility group box 1
HPV: Human papillomavirus
Hsp60: Heat shock protein 60
ICAT: Inhibitor of β-catenin and T-cell factor
Kac: Lysine acetylation
KAT: Lysine acetyltransferase
Kla: Lysine lactylation
LDH: Lactate dehydrogenase
LGSH: Lactoylglutathione
MCT: Monocarboxylate transporter
ME2: Malate enzyme 2
MI/MII: Metaphase I/II
MRE11: Meiotic recombination 11
NAA10: N-alpha-acetyltransferase 10
NAD^+^: Nicotinamide adenine dinucleotide
NADH: Reduced nicotinamide adenine dinucleotide
NADPH: Reduced nicotinamide adenine dinucleotide phosphate
NBS1: Nijmegen breakage syndrome 1
NF-κB: Nuclear factor-kappa B
NHE7: Solute carrier family 9 member A7
NLRP3: NLR family pyrin domain-containing 3
NRF: Nuclear respiratory factor
PCOS: Polycystic ovary syndrome
PDC: Pyruvate dehydrogenase complex
PDH: Pyruvate dehydrogenase
PDHA1: Pyruvate dehydrogenase E1 subunit alpha 1
PDK: Pyruvate dehydrogenase kinase
PD-L1: Programmed death ligand 1
PFKP: Phosphofructokinase platelet type
PE: Preeclampsia
PFK: Phosphofructokinase
PFKM: Phosphofructokinase muscle type
PGK1: Phosphoglycerate kinase 1
PHD: Plant homeodomain
PGC-1α: Peroxisome proliferator-activated receptor gamma coactivator 1-alpha
PK: Pyruvate kinase
PKA: Protein kinase A
PKM2: Pyruvate kinase M2
POI: Primary ovarian insufficiency
PPP: Pentose phosphate pathway
PRDX6: Peroxiredoxin 6
PrE: Primitive endoderm
PRMT1: Protein arginine methyltransferase 1
PTM: Post-translational modification
RASD2: Ras homolog enriched in striatum 2
RIF: Recurrent implantation failure
ROS: Reactive oxygen species
RPL: Recurrent pregnancy loss
SCD1: Stearoyl-CoA desaturase 1
SESN2: Sestrin 2
SLC9A7: Solute carrier family 9 member A7
SIRT: Sirtuin
SNAP29: Synaptosomal-associated protein 29
TAMs: Tumor-associated macrophages
TCA: Tricarboxylic acid
TME: Tumor microenvironment
TOX: Thymocyte selection-associated high mobility group box
USP39: Ubiquitin specific peptidase 39
VDAC1: Voltage-dependent anion channel 1
VHL: Von Hippel–Lindau
ZGA: Zygotic genome activation
