# Correction: Research progress on protein lactylation in female reproductive disease: molecular mechanisms, functions, and therapeutic implications

**DOI:** 10.3389/fphar.2026.2007319

**Published:** 2026-10-15

**Authors:** Xiaoyang Shen, Yan Chen, Hongxia Deng, Nannan Zhang, Liangzhi Xu

**Affiliations:** 1 Reproductive Endocrinology and Regulation Laboratory, West China Second University Hospital, Sichuan University, Chengdu, China; 2 Department of Obstetrics and Gynecology, West China Second University Hospital, Sichuan University, Chengdu, China; 3 Key Laboratory of Birth Defects and Related Diseases of Women and Children (Sichuan University), Ministry of Education, Chengdu, China; 4 National Center for Birth Defect Monitoring, West China Second University Hospital, Sichuan University, Chengdu, China

**Keywords:** epigenetic regulation, female reproductive diseases, gynecological tumors, lactate, lactylation, post-translational modification, therapeutic target

There was a mistake in Section **3 Lactylation in the female reproductive system: molecular mechanisms and clinical implications**, *3.1.3 Lactylation and embryonic development*, paragraph 1. The opening words of the first sentence were incorrectly published as “Preeclampsia (PE) embryonic development” instead of “Preimplantation embryonic development”. The corrected sentence reads:

“Preimplantation embryonic development spans from fertilization to blastocyst implantation into the uterine endometrium around day 7.”

There was a mistake in Section **3 Lactylation in the female reproductive system: molecular mechanisms and clinical implications**, *3.2.1 Lactylation and preeclampsia*, paragraph 1. The abbreviation “PE” was omitted after the first occurrence of “Preeclampsia”. The corrected sentence reads:

“Preeclampsia (PE), a pregnancy-specific hypertensive disorder caused by placental dysfunction, is one of the main causes of maternal and perinatal morbidity and mortality globally.”

There was a typo in Section **3 Lactylation in the female reproductive system: molecular mechanisms and clinical implications**, *3.2.3 Lactylation and endometrial receptivity*, paragraph 4. “In” was repeated twice at the beginning of the paragraph. The extra “in” has been removed.

There was a mistake in Section **3 Lactylation in the female reproductive system: molecular mechanisms and clinical implications**, *3.5.2 Cervical cancer*, paragraph 2. The wording indicating that the mechanism increases NADPH and GSH levels was inadvertently omitted when “NADPH” was expanded. The corrected sentence reads:

“This increases the levels of reduced nicotinamide adenine dinucleotide phosphate (NADPH) and GSH, decreases ROS, and promotes cervical cancer cell proliferation, migration, invasion, and xenograft growth (Meng et al., 2024a).”

The original version of this article has been updated.

